# Unveiling the Complexities of Medications, Substance Abuse, and Plants for Recreational and Narcotic Purposes: An In-Depth Analysis

**DOI:** 10.3390/pharmacy13010007

**Published:** 2025-01-22

**Authors:** Iasmina-Alexandra Predescu, Alex-Robert Jîjie, Dalia Pătraşcu, Aida-Luisa-Vanessa Pasc, Elisaveta-Ligia Piroş, Cristina Trandafirescu, Cristian Oancea, Cristina Adriana Dehelean, Elena-Alina Moacă

**Affiliations:** 1 Discipline of Toxicology, Drug Industry, Management and Legislation, Faculty of Pharmacy, “Victor Babeş” University of Medicine and Pharmacy Timisoara, 2nd Eftimie Murgu Square, 300041 Timisoara, Romania; iasmina-alexandra.predescu@umft.ro (I.-A.P.); alex-robert.jijie@umft.ro (A.-R.J.); aida-luisa.pasc@student.umft.ro (A.-L.-V.P.); cadehelean@umft.ro (C.A.D.); alina.moaca@umft.ro (E.-A.M.); 2 Research Center for Pharmaco-Toxicological Evaluation, “Victor Babes” University of Medicine and Pharmacy Timisoara, 2nd Eftimie Murgu Square, 300041 Timisoara, Romania; 3 Faculty of Medicine, “Vasile Goldiş” Western University of Arad, 86 Liviu Rebreanu Street, 310048 Arad, Romania; piros.ligia@uvvg.ro; 4 Discipline of Pharmaceutical Chemistry, Faculty of Pharmacy, “Victor Babes” University of Medicine and Pharmacy Timisoara, 2nd Eftimie Murgu Square, 300041 Timisoara, Romania; trandafirescu.cristina@umft.ro; 5 Discipline of Pneumology, Faculty of Medicine, “Victor Babeş” University of Medicine and Pharmacy Timisoara, 2nd Eftimie Murgu Square, 300041 Timisoara, Romania; oancea@umft.ro

**Keywords:** substance abuse, medication abuse, recreational plants, narcotic plants, substance use disorders, psychoactive substances, addiction

## Abstract

The complexities surrounding the use of medications, substance abuse, and the recreational use of plants are multifaceted and warrant a comprehensive examination. This review highlights the complexities surrounding the consumption of chemical substances in excess or for non-medical purposes, obtained through legal prescriptions, over-the-counter purchases, or illicit means, with an emphasis on the predictive role of stressors and individual-level variables in the development of substance use disorders, as well as the influence of the regulatory environment on patterns of consumption. Additionally, the alarming escalation in the mortality rate associated with illicit drug and opioid overdoses is also underscored. The recreational use of prescription medications can lead to significant health risks, particularly when combined with other substances; therefore, the need for interventions and preventive measures to address substance abuse among various populations is imperative. Furthermore, novel insights on substance abuse addiction, exploring the neurobiological mechanisms underlying addiction, and discussing treatment approaches and interventions are elucidated. Advancements in technology for detecting substance abuse are also highlighted, displaying innovative tools for more effective identification and monitoring. In conclusion, the complexities of medications, substance abuse, and the recreational use of plants reveal a landscape marked by overlapping motivations and health implications. The distinction between medical and recreational use is critical for understanding user behavior and addressing public health concerns.

## 1. Introduction

Substance abuse is a significant issue with far-reaching consequences, such as damage to all organs and especially brain development in adolescents. It contributes to rising mortality rates, an increase in crimes committed while acquiring drugs, low employment rates, higher risks of poverty, and damaged relationships with family, friends, and loved ones [1,2].

Defined as the misuse of psychoactive substances for non-medicinal purposes, substance abuse poses a considerable medical problem [1]. Studies have shown that stressors and individual-level variables play a predictive role in the development of substance use disorders [3].

Medication and substance abuse entails the consumption of chemical substances in excessive amounts or for non-medical purposes, whether obtained through legal prescriptions, over-the-counter purchases, or illicit means. The recreational use of substances is often linked to the regulatory environment surrounding their availability, influencing patterns of consumption [4,5]. Addiction, a hallmark of substance abuse, is typified by the loss of control over substance intake and disregard for adverse outcomes, constituting a medical disorder characterized by the compulsive pursuit of psychoactive agents, indicative of a neuropsychiatric condition [6,7].

Substance abuse presents a significant global challenge. Recent decades have witnessed a notable surge in the availability of drugs, prompting widespread concern among the populace and posing a substantial risk to public health [8]. The substances implicated in this issue encompass a range, including cocaine, cannabis, tobacco, and opioids such as heroin, codeine, and morphine. These substances fall into two broad categories: legal and illegal. Cocaine and heroin are classified as illicit substances, while cannabis straddles both categories due to variations in its legal status across different nations. Tobacco enjoys legal approval for consumption worldwide, and opioid medications are legally sanctioned for managing chronic pain conditions [9,10].

The use of medications and substances, particularly those derived from plants for recreational and narcotic purposes, is a topic of significant concern and interest in both medical and societal contexts. One of the most known plants throughout history for use in both medicinal and recreational purposes is *Cannabis sativa* [11]. The psychoactive properties of Cannabis, particularly the cannabinoid THC, have made it the subject of extensive research [12]. The diverse array of phytochemicals present in Cannabis, including cannabinoids, terpenes, and phenolic compounds, have been studied for their potential medicinal benefits [13]. Additionally, the genetic structure of Cannabis has been explored to understand the differences between drug types like marijuana and hemp [12].

The mortality rate associated with illicit drug and opioid overdoses has exhibited a steady escalation over the past two decades. Consequently, in 2021, there was an estimated surge of over 90% in fatalities as opposed to the statistics from 1999. Furthermore, empirical evidence underscores that individuals of the male sex constitute a predominant proportion of overdose incidents [14].

Substance abuse, encompassing the misuse of legal and illicit substances, is a global issue that affects various populations [15]. Studies have shown that substances like alcohol, tobacco, and illegal drugs are commonly abused among the population, highlighting the need for interventions and preventive measures [16]. Understanding the motivations behind substance abuse, whether for recreational purposes or to cope with stress, is crucial for developing effective interventions [17]. Motives for the medical misuse of prescription opioids among adolescents have been linked to an increased risk of substance abuse, emphasizing the complex interplay between medical prescription practices and substance misuse [18]. The misuse and abuse of substances like benzodiazepines, often in combination with other drugs like opioids and alcohol, pose significant challenges in terms of addiction and treatment [19].

An escalation in substance abuse was noted, with approximately 1.7 million individuals in the United States affected by addiction or disorders related to opioid substances by 2017. In Norway, a decline in these figures was observed. Yet, the reduction in fatalities was predominantly associated with the misuse of prescription painkillers rather than heroin (illicit drugs), underscoring the persistent societal challenges posed by opioids [20]. Cocaine misuse, in contrast, resulted in a higher number of overdose fatalities, with emergency departments recording 70,237 overdose-related deaths in 2017 [21]. Despite being the third most abused substance globally, cannabis is often underestimated by consumers who believe they do not develop addictive behaviors or withdrawal symptoms post-consumption [22]. Tobacco, acknowledged for its harmful effects, was consumed by approximately 18% of the global population in 2013, with projections indicating a continued rise in consumption rates by 2030 [23,24]. Smoking has become a prevalent practice among both adults and youth, with statistics revealing that 80.2% of high school students have experimented with tobacco products, and 11.5% of adults were regular smokers in 2021 [25]. Furthermore, a significant proportion of adolescent smokers initiated tobacco use at a young age, between 10 and 12 years old [26].

In the context of substance abuse prevention, education plays a vital role in equipping medical professionals with the knowledge and skills to address substance-related disorders in their practice. Courses related to drug abuse prevention in medical curricula are essential for raising awareness and promoting effective interventions [27]. Novel interventions in the realm of substance abuse and addiction have been sanctioned to mitigate usage. In pain management, the emphasis lies on administering opioids at a minimally effective dose [28]. Innovations in tobacco and nicotine products focus on developing less deleterious alternatives to diminish the morbidity and mortality associated with cigarettes [29]. Regulatory measures such as taxation increments and public consumption restrictions are pivotal in curtailing the sale and utilization of these substances [30,31]. Specialized initiatives are being implemented to deter illicit substance use. Diverse therapeutic modalities play a pivotal role in cessation efforts and preventing relapse [31,32].

The purpose of the review is to provide updated information and novel perspectives on the use of various plants for recreational and narcotic purposes. The scope of the review encompasses an examination of the pharmacological properties, potential risks, and societal implications associated with the consumption of these plants. By synthesizing current research findings, the review aims to contribute to a better understanding of the complex relationship between medication, substance abuse, and the use of plants for recreational and narcotic purposes.

## 2. History of Medication, Substance Abuse, and Plants for Recreational and Narcotic Purposes

### 2.1. Early Use of Plants for Recreational and Narcotic Purposes

The utilization of psychoactive plants for ceremonial and recreational purposes has been a common practice throughout history. Some African Helichrysum species, although containing non-narcotic compounds, have been used for ritual inebriating fumigations [33]. The Mandrake plant, known for its effects and compounds, has been associated with healing, inducing madness, and even causing death [34]. The early relationship between humans and psychoactive plants, whether for medicinal, ritualistic, or recreational purposes, highlights the long-standing history of plant usage for altering states of consciousness [35].

Various cultures have acknowledged specific plants’ sacred attributes, employing them for recreational and therapeutic purposes. For example, Indigenous communities have traditionally embraced coca leaves for their stimulating effects, with documented instances of leaf-chewing dating back to 3000 years ago [6]. In the 19th century, cocaine was extracted from these leaves and utilized as a stimulant in beverages and analgesic solutions. Presently, cocaine is available in the forms of cocaine hydrochloride or free base, typically as a white powder for intravenous, intranasal, or oral consumption or as a solid mass for smoking, commonly referred to as “crack cocaine” [36].

The Sumerians utilized the sap of the poppy plant for therapeutic purposes [6]. Opium’s medicinal properties were recognized before the identification of morphine. In antiquity, the Egyptians harnessed opium’s analgesic and mood-enhancing effects [37]. The isolation of morphine from the opium poppy in 1803 significantly influenced pain management. The discovery of morphine and codeine spurred advancements in synthesizing opioid alkaloids for pain relief [38]. Heroin, derived from morphine through acetylation, initially transformed pain management practices and was widely manufactured for its efficacy in treating respiratory ailments. However, its addictive properties were soon uncovered, leading to its classification as a substance of abuse [39].

Recent paleobotanical research indicates that cannabis has been utilized for approximately 11,700 years, with archaeological evidence pinpointing its presence in the Altai Mountains region of Central Asia. Initially harnessed for practical purposes such as rope and net production, cannabis also served as a dietary staple and a source of oil from its seeds. The serendipitous discovery of its psychoactive properties led to its widespread cultivation and dissemination globally [40]. Scientific investigations have revealed the therapeutic potential of cannabis in alleviating conditions like headaches, rheumatic pains, insomnia, constipation, and venereal diseases [41]. The evolution of cannabis consumption has progressed alongside the development of THC-containing products, with smoking being the traditional method. Modern consumption methods include manual rolling, using filtering devices like bongs, vaping, and ingesting THC through edibles such as brownies and teas [42].

Tobacco, a plant with a long history of recreational use dating back to ancient times, has been integral to ceremonies and rituals within North American tribes for over 2300 years. While its consumption traditionally lacked significant psychoactive effects, the cultivation of tobacco held sacred significance, influencing both the tribe and its initiates. Gender roles also played a role in the cultivation of tobacco, with some cultures restricting women from engaging in this practice. Over time, the method of consuming tobacco evolved from smoking it in specialized “T”-shaped pipes to the development of modern products like cigarettes, cigars, and electronic cigarettes [43]. The emergence of electronic cigarettes in 2007 marked a significant shift in tobacco consumption, utilizing a liquid solution containing nicotine and artificial flavors that is vaporized through heating. This innovation has since progressed to include disposable electronic cigarettes, commonly referred to as vapes, as well as more advanced devices designed for heating tobacco specifically. Additionally, alternative nicotine and tobacco products such as nicotine gum, sprays, and patches have been introduced as part of smoke-free initiatives [44].

The historical use of plants for recreational and narcotic purposes is a fascinating aspect of human civilization. From cannabis and opium poppy to psychoactive plants like the Mandrake, the relationship between humans and plants for altering consciousness dates back millennia. The diverse phytochemical composition of these plants, including cannabinoids, alkaloids, and terpenes, has contributed to their medicinal, ceremonial, and recreational significance throughout history. Understanding the historical context of plant usage for recreational and narcotic purposes provides valuable insights into the cultural, social, and pharmacological dimensions of these practices.

### 2.2. Evolution of Law and Regulations

The evolution of laws and regulations concerning medication, substance abuse, and plants for recreational and narcotic purposes has been a complex and dynamic process influenced by various factors such as societal attitudes, scientific research, and political considerations. The laws and rules for the consumption of substances of abuse vary both regionally and depending on the substance incriminated. In recent years, there has been a notable shift in the legislation governing substances like cannabis, with many jurisdictions relaxing restrictions on cultivation and use for research, medicinal, and even recreational purposes [12]. This change reflects a growing acceptance of the potential therapeutic benefits of substances like marijuana, which has been traditionally used for both recreational and medicinal purposes [45].

The liberalization of cannabis policies in numerous U.S. states and countries like Spain, Uruguay, and Portugal over the past two decades has been a significant development in drug regulation [46]. This shift toward decriminalization and legalization for medical and recreational use signifies a departure from the previous prohibitionist approach that classified cannabis as a narcotic and banned its use [47]. As geographic regulations and testing guidelines for cannabis products continue to evolve, there is a need for comprehensive frameworks to ensure the safety and quality of these substances [48].

The recreational use of cannabis has a historical background, dating back to its recognition as a medicinal substance in 1850 when it was included in the American Pharmacopeia. However, due to widespread abuse, cannabis was classified as an illegal drug in 1970, leading to its possession being deemed a misdemeanor [41]. Presently, medical cannabis enjoys legal status in numerous countries across the United States and Europe. The trend toward decriminalization of cannabis use has been on the rise since 2012, primarily driven by the reduction in penalties associated with personal consumption [49]. Notably, the legalization of recreational cannabis has been implemented in various countries, including Canada and 15 states within the United States, as well as in European nations such as Germany, Spain, and the Netherlands [50].

The 20th century marked the inception of significant growth in the cigarette industry. In response to mounting anti-smoking movements, cigarette manufacturing faced prohibition and stringent oversight in 15 states initially. Subsequently, several states enacted more stringent regulations concerning tobacco marketing and smoking in public spaces. Major tobacco corporations devised strategies aimed at refuting the harmful effects of cigarettes. The official acknowledgment of the correlation between smoking and lung cancer in 1964 precipitated the enactment of the Federal Cigarette Labeling and Advertising Act of 1965 [51]. This legislation mandated that cigarette manufacturers provide consumers with explicit warnings about the hazards associated with smoking. Complementing these efforts, the 2009 Family Smoking Prevention and Tobacco Control Act prohibited the advertisement of cigarettes and cigars through mass media [52]. To mitigate the health repercussions of smoking, tobacco levies are periodically increased [30].

Opioids, a class of drugs utilized for pain management, straddle the fine line between therapeutic use and potential misuse. The responsibility for the overprescription of opioids primarily falls on healthcare professionals. The Controlled Substances Act of 1970 was enacted to regulate the manufacturing and distribution of opioids, aiming to mitigate instances of abuse [53]. States grappling with escalating rates of opioid abuse and overdose have responded by enacting stringent legislation governing prescription practices. These regulations encompass establishing centralized databases to monitor controlled substance dispensation and imposing restrictions on the prescribing authority of medical practitioners. As of 2019, 38 states had implemented such laws, which also include limitations on prescription quantities, varying based on individual state consumption patterns [54].

Cocaine, with a well-documented historical trajectory, was initially employed as an anesthetic within the medical field toward the conclusion of the 19th century. Its medicinal application promptly brought to light the peril of addiction among patients. After the adverse occurrences during that era, cocaine was classified as a controlled substance under the Misuse of Drugs Act of 1971 [55]. Despite the implementation of measures aimed at curtailing illicit substance abuse, the United States government introduced novel regulations on the supervision of substance distribution. Presently, four enduring regulations are in effect, specifically targeting the prevention of illicit drug manufacturing, notably focusing on cocaine and heroin [56].

The evolution of laws and regulations concerning medication, substance abuse, and plants for recreational and narcotic purposes is a multifaceted process influenced by scientific advancements, societal attitudes, and public health considerations. The shift toward liberalizing cannabis policies in various jurisdictions reflects changing perceptions of these substances and their potential benefits. However, challenges such as stigmatization, product differentiation, and ensuring safety and quality remain critical aspects of regulatory frameworks in this evolving landscape.

### 2.3. Impact of Medication and Substance Abuse on Society

The impact of medication, substance abuse, and plants for recreational and narcotic purposes on society is a multifaceted issue that encompasses various aspects of human behavior, health, and culture. The abuse of substances like cocaine, derived from plants like *Erythroxylum coca*, has had significant repercussions on society, leading to addiction, health problems, and social issues [57].

In contemporary society, there is a growing trend, particularly among younger generations, to experiment with hallucinogenic plants for recreational purposes [58]. This trend raises concerns about the accessibility and potential risks associated with the use of these substances. Additionally, the socioeconomic context plays a significant role in shaping patterns of substance abuse, with factors such as tobacco, alcohol, and cannabis use among young adults being influenced by social and economic conditions. Understanding the societal implications of substance abuse requires a comprehensive examination of the interplay between individual behavior, cultural norms, and economic disparities [59].

Plants have long been utilized for their medicinal properties, with many traditional pharmacopeias incorporating various plant species for therapeutic purposes. The exploitation of plant ingredients for anesthetic preparations, as seen in historical formulations, underscores the diverse roles that plants have played in society, ranging from narcotic effects to adjuvant functions [60]. However, the use of certain plants, such as kratom (*Mitragyna speciosa*), has raised concerns due to their addictive properties and potential adverse effects on individuals and communities. The regulation of plants with narcotic properties reflects the ongoing dilemma authorities face in balancing public health concerns with individual freedoms and cultural practices [61].

The misuse of substances has been demonstrated in contemporary research to exert a negative influence on both professional and social relationships. Such consumption is linked to a decline in the individual’s motivation, resulting in the neglect of routine responsibilities. This behavior may lead to severe repercussions such as unemployment, significant financial implications, a deterioration in interpersonal connections, and a decline in overall quality of life [62].

The etiology of substance addiction encompasses pathological, familial, genetic, and individual developmental factors. The onset of substance use at a young age correlates with heightened addiction susceptibility. Recent studies identify depression and ADHD as additional risk factors in conjunction with the variables above. Childhood psychological traumas exert a substantial influence on individual development, thereby elevating the vulnerability of this demographic to substance abuse [63].

Despite the association between marijuana legalization and increased consumption, recent data indicate that the patterns of heightened usage were observable before legalization. In Canada, studies conducted within 2 years pre- and post-legalization present varying viewpoints on the prevalence of cannabis use and the well-being of users. Moreover, it is recommended that more comprehensive post-consumption evaluations be conducted at intervals of 2–3 years [64,65]. In contrast to the legalization of cannabis, a survey involving allergists revealed that 30% of physicians do not inquire about patients’ potential cannabis use, indicating that this topic remains stigmatized within certain segments of the population [65].

Tobacco, ranking second among substances of abuse, exhibits distinct consumption patterns within the population. Research indicates a higher prevalence of consumption among men, with a tendency toward higher daily intake (exceeding one package per day) compared to women. The habit of smoking is often linked to alcohol use, poor dietary choices, and, in some instances, a sedentary lifestyle [66]. Among adolescents, cigarette consumption is associated with exposure to secondhand smoke, social circles, and disadvantaged socioeconomic backgrounds [26]. Studies have illustrated the profound influence of marketing strategies on consumer behavior. Neuroimaging investigations have revealed that novel cigarette packaging designs stimulate neural pathways associated with reward processing and cognitive regulation. Enhancements in product quality, cigarette design, and packaging aesthetics have been shown to boost sales and user consumption rates. Furthermore, the sensory gratification derived from smoking may stem from the ritualistic nature of the activity [67].

Chronic pain often drives the widespread consumption of opioids, with the rapid development of tolerance in affected individuals [68]. The progression of pain pathology can result in comorbid conditions such as anxiety and depression among patients. In response to the adverse effects of opioids, such as sedation, individuals may escalate their intake to manage their symptoms [69]. Additionally, some patients may underestimate the risks associated with opioids due to their prescription status, leading to misuse or sharing of medication with others. This misconception can contribute to the rapid onset of addiction, culminating in a concerning rise in overdose and mortality rates within society [70].

The compulsive use of cocaine is categorized as a psychiatric disorder known as cocaine use disorder, impacting a substantial portion of the global population and posing a societal concern [71]. This disorder may present through instances of physical aggression that foster and escalate criminal activities and violence [72]. Regrettably, there is a notable escalation in cocaine trafficking, leading to a rise in consumption rates. Despite endeavors to eliminate the illicit cocaine market, it endures, concurrently perpetuating violence and exerting adverse effects on public health [73].

## 3. Most Commonly Used Plants for Recreational and Narcotic Purposes Nowadays

Plants containing psychoactive compounds have a long history of diverse uses, ranging from medicinal and spiritual to recreational purposes, evolving toward more recreational and narcotic applications, notably seen in the legalization of cannabis in multiple jurisdictions [74]. This shift is not limited to cannabis but extends to other plants like opium poppy, ayahuasca, kratom, and peyote, reflecting an increasing trend in their consumption [75]. The availability and accessibility of these plants have been enhanced by technological advancements and globalization, making them more obtainable than ever before. The surge in plant consumption for recreational and narcotic purposes is influenced by factors such as legalization, which impacts pricing, potency, and availability [74]. The potential therapeutic value of these plants, particularly those with hallucinogenic properties, is being increasingly explored for novel psychotropic drug development [76].

The recreational use of plants, particularly those with psychoactive properties, can be attributed to various reasons that cater to different individual needs and desires. Some individuals engage in the recreational use of plants solely to seek euphoric and perception-altering experiences, aiming to escape from the constraints of reality [77]. This desire for altered states of consciousness is a driving force behind the recreational use of psychoactive plants, as they offer a reprieve from the mundane aspects of everyday life. Additionally, people turn to these plants as a coping mechanism for stress, anxiety, and other mental health issues, believing that the psychoactive compounds present in these plants can provide a momentary escape from their problems, offering a sense of relaxation and relief [78].

Moreover, another significant reason for the recreational use of plants is the pursuit of self-exploration, with individuals seeking to gain a deeper understanding of themselves and the world around them. Through the consumption of psychoactive herbs, some individuals embark on journeys of introspection and self-discovery, using these plants as tools to explore their consciousness and emotions. Studies have indicated that individuals engaging in self-discovery through psychoactive herbs often report feelings of spiritual connectedness and enhanced psychological well-being. This introspective aspect of plant use highlights a more profound motivation beyond mere recreation, delving into the realms of personal growth and enlightenment [79].

Certain plants’ traditional and cultural significance also plays a role in their recreational use. For instance, the South Pacific medicinal plant kava (*Piper methysticum*) has been historically utilized for its relaxant properties. It is now incorporated into modern phytotherapy as a treatment for anxiety [78]. This cultural heritage surrounding the use of specific plants adds a layer of depth to their recreational consumption, intertwining traditional practices with contemporary recreational preferences. Additionally, the recreational use of plants is often intertwined with societal behaviors and norms, shaping human culture in multifaceted ways. The consumption of psychoactive plants can lead to habit formation and elicit various effects such as relaxation, stimulation, pain relief, enhanced memory, and reduced anxiety, reflecting the complex interplay between plant use and cultural practices [80].

Furthermore, the recreational use of plants is not solely driven by individual motivations but is also influenced by external factors such as accessibility and societal acceptance. The surge in recreational cannabis use among adolescents and adults has prompted a closer examination of the short-term and long-term effects of consuming this plant on both the mind and body [81]. This shift in societal attitudes toward cannabis has led to increased scrutiny and research into its recreational use, highlighting the evolving landscape of plant consumption in contemporary society. Additionally, the availability of specific plants for recreational purposes, such as those used in making recreational teas, underscores the continued relevance of traditional practices in modern recreational settings. Despite changes in plant usage over time, some recreational teas remain popular and are even served in commercial establishments, showcasing the enduring appeal of plant-based recreational experiences [82].

The use of serotonergic hallucinogens, such as LSD, has been documented for recreational, personal, spiritual, and therapeutic purposes, indicating the multifaceted nature of plant-based psychoactive substances [83]. Similarly, ayahuasca (*Banisteriopsis caapi*), traditionally used for spiritual and medicinal purposes, has seen a shift toward recreational use, particularly in Western societies, underscoring the evolving trends in plant consumption [84]. Kratom (*Mitragyna speciosa*), another plant with psychoactive properties, has gained popularity as a recreational drug globally, reflecting a broader pattern of increased recreational plant use [85]. These shifts in usage patterns raise important questions about the implications of widespread recreational plant consumption and its potential risks.

Research on ayahuasca (*Banisteriopsis caapi*) has suggested potential therapeutic uses for treating psychiatric disorders and addictions, pointing toward the complex interplay between traditional medicinal practices and modern therapeutic approaches [86]. Additionally, studies on kratom (*Mitragyna speciosa*) have highlighted its interaction with various receptors in the body, leading to a wide range of effects and shedding light on its emergence within the broader context of substance use disorders [87]. The safety and potential benefits of ayahuasca use under certain conditions have been indicated, emphasizing the need for a nuanced understanding of the risks and benefits associated with plant-based psychoactive substances [88]. Moreover, the increasing clinical awareness around kratom use and the assessment of kratom use disorder among United States adults underscore the growing recognition of the impact of these plants on public health [89].

The global phenomenon of ayahuasca use and the recreational consumption of DMT (*N*,*N*-dimethyltryptamine) and similar alkaloids have raised concerns about uncontrolled use and the potential for severe intoxications, highlighting the need for regulatory measures and public health interventions [90]. The detection and quantification of psychoactive compounds in plant matrices have become essential for monitoring and addressing the recreational abuse of plants like kava, emphasizing the importance of analytical techniques in understanding and regulating plant-based psychoactive substances [91]. Furthermore, the toxicological issues related to kratom usage underscore the complexities surrounding the management of substance abuse disorders and withdrawal effects, necessitating a comprehensive approach to address these challenges [92].

The evolving landscape of plant consumption for recreational and narcotic purposes presents a complex interplay between traditional uses, modern trends, regulatory frameworks, and public health considerations. Understanding the multifaceted nature of plant-based psychoactive substances, their potential benefits, risks, and societal implications is crucial for developing evidence-based policies and interventions to address the challenges posed by the increasing use of these plants [93]. The recreational use of plants for various purposes, including seeking altered states of consciousness, coping with mental health issues, self-exploration, and cultural practices, reflects the diverse motivations and influences that shape human interactions with psychoactive flora. From traditional herbal remedies to contemporary recreational preferences, the consumption of plants for recreational purposes is a multifaceted phenomenon that intertwines individual desires, cultural heritage, and societal norms. Understanding the reasons behind the recreational use of plants provides insights into human behavior, cultural practices, and the intricate relationships between individuals and the natural world [94,95].

By synthesizing research findings and empirical evidence, this chapter provides a comprehensive overview of the most used plants for recreational and narcotic purposes, shedding light on their historical significance, contemporary trends, and future implications for public health and regulatory frameworks.

**Figure 1:**
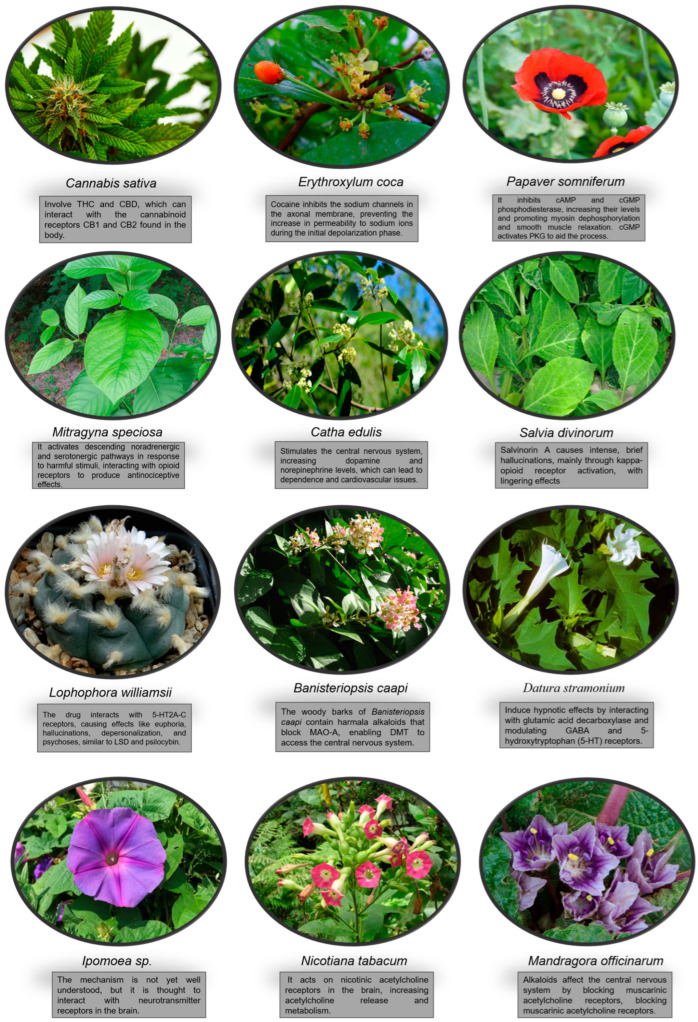
Mechanism of action for plants used today for recreational and narcotic purposes: *Cannabis sativa* (https://www.countynewscenter.com/new-cannabis-program-to-address-inequity-in-unincorporated-area/, accessed on 4 January 2025), *Erythroxylum coca* (https://www.fieldmuseum.org/collection/erythroxylum, accessed on 4 January 2025), *Papaver somniferum* (https://ro.wikipedia.org/wiki/Mac_de_gr%C4%83din%C4%83, accessed on 24 November 2024), *Mitragyna speciosa* (https://en.wikipedia.org/wiki/Mitragyna_speciosa, accessed on 4 January 2025), *Catha edulis* (https://www.inaturalist.org/taxa/190367-Catha-edulis, accessed on 4 January 2025), *Salvia divinorum* (https://www.iceers.org/salvia-divinorum-basic-info/, accessed on 24 November 2024), *Lophophora williamsii* (https://llifle.com/Encyclopedia/CACTI/Family/Cactaceae/1117/Lophophora_williamsii, accessed on 4 January 2025), *Banisteriopsis caapi* (https://ntbg.org/database/plants/detail/banisteriopsis-caapi, accessed on 24 November 2024), *Datura stramonium* (https://www.infoflora.ch/de/flora/datura-stramonium.html, accessed on 4 January 2025), *Ipomoea* sp. (https://www.britannica.com/plant/Ipomoea, accessed on 24 November 2024), *Nicotiana tabacum* (https://www.nzpcn.org.nz/flora/species/nicotiana-tabacum/, accessed on 4 January 2025), and *Mandragora officinarum* (https://www.britannica.com/plant/Mandragora-officinarum, accessed on 24 November 2024).

Figure 1 illustrates the mechanisms of action of plants used for recreational and narcotic purposes. This information enhances our understanding of how they exert their effects and the risks associated with exposure to them.

### 3.1. Cannabis sativa (Cannabis)

#### 3.1.1. Historical Background

*Cannabis sativa*, commonly known as marijuana, has a long and complex history intertwined with human civilization. It belongs to the Cannabaceae family and is native to Central Asia [13]. It is an annual flowering herb that can be classified into two main species: *Cannabis sativa* var. sativa, which is taller and more fibrous, and *Cannabis sativa* var. indica, which is shorter and more psychoactive [96]. The distinction between these two types of cannabis and the different biological effects associated with them has been a subject of significant interest and research [97]. *Cannabis sativa* has been cultivated for centuries for various purposes, including medicinal, recreational, and industrial uses. The plant’s versatility is evident in its applications, ranging from medicine to textile fiber production and even religious rituals [98]. The use of *Cannabis sativa* for recreational and narcotic purposes has been a topic of debate and study for many years. The plant contains a wide array of compounds, with over 500 identified in the Cannabis genus, distributed across 18 chemical classes [99]. Among these compounds, the presence of high concentrations of cannabinoids, such as delta-9-tetrahydrocannabinol (THC) and cannabidiol (CBD), in Cannabis flowers play crucial roles in the plant’s effects on the human body [100].

#### 3.1.2. Drug Policy

The legalization of *Cannabis sativa* for various purposes, including medical and recreational use, has been a significant trend in recent years. In the United States, the 2014 Farm Bill paved the way for the re-introduction of hemp (*Cannabis sativa* L.) as an industrial crop [101]. Furthermore, the Agriculture Improvement Act of 2018 legalized the cultivation of *Cannabis sativa* with a THC content of no more than 0.3%, opening up new opportunities for the production of hemp-derived products. This shift in legislation reflects changing attitudes toward *Cannabis sativa* and its potential economic and agricultural benefits [102].

#### 3.1.3. Pharmacological Properties and Health Implications

The pharmacological properties of *Cannabis sativa* have been the subject of extensive research, with studies exploring its effects on various medical conditions. The plant has been used for centuries for recreational purposes and, more recently, in the treatment of patients with neurological or psychiatric disorders [103]. The potential therapeutic uses of *Cannabis sativa* have led to investigations into its efficacy in managing conditions such as chronic pain, postoperative discomfort, and substance-related disorders [104]. The diverse chemical composition of *Cannabis sativa*, including cannabinoids, terpenes, and other compounds, contributes to its pharmacological effects and potential medical applications [105].

Despite its recreational appeal, the consumption of *Cannabis sativa* is associated with adverse effects, mainly when used in excess or by vulnerable populations. Short-term adverse effects of cannabis use can include psychotic states, cognitive impairments, and dependence, particularly among regular users [106]. Furthermore, the co-use of cannabis with other substances, such as tobacco, can exacerbate dependence and have implications for overall health and well-being. Understanding the risks associated with cannabis consumption is crucial for developing effective public health interventions and harm reduction strategies [107].

#### 3.1.4. Mechanism of Action

Cannabis acts through the activation of cannabinoid receptors, in particular, the CB1 and CB2 receptors of the endocannabinoid complex system. Interaction with presynaptic CB1 receptors on GABAergic inhibitory neurons in the reward pathway inhibits adenylate cyclase, reducing electrical excitation and neurotransmitter release. This inhibition of GABA release causes a disinhibition of dopaminergic neurons, leading to increased synaptic dopamine levels—a mechanism similar to that of opioids. The main active compound in cannabis, THC, is primarily responsible for the plant’s psychoactive properties (which produce a euphoric and relaxing state). At the same time, CBD is known for its potential therapeutic benefits [108].

The narcotic and recreational effects of *Cannabis sativa* are primarily attributed to cannabinoids and terpenoids. These bioactive molecules interact with the endocannabinoid system in the human body, influencing various physiological processes and producing psychoactive effects [109]. Consumers seek marijuana for its recreational effects, as it can induce pleasurable states, enhance sensory perception, distort time perception, and facilitate social interactions. The levels and ratios of these cannabinoids in *Cannabis sativa* determine its potency and effects, making it a subject of interest for both recreational users and researchers exploring its medicinal potential [110].

#### 3.1.5. Future Perspectives on Public Health

Looking toward the future, the implications of *Cannabis sativa* for public health are multifaceted. The increasing consumption of cannabis for recreational purposes globally raises concerns about the potential impact on mental health and substance use disorders. With approximately 200 million cannabis users worldwide, monitoring trends in consumption, addressing regulatory challenges, and promoting evidence-based education are essential for mitigating potential harms [111]. Additionally, as research on *Cannabis sativa* advances, there is a need to explore its therapeutic potential while ensuring safe and responsible use in the context of evolving legal frameworks [112].

#### 3.1.6. General Conclusion

Overall, *Cannabis sativa* remains a complex and multifaceted plant with a long history of use for recreational, medicinal, and industrial purposes. Understanding its historical significance, contemporary consumption trends, mechanisms for narcotic and recreational purposes, chemical compounds responsible for effects, adverse effects of consumption, and future implications for public health is essential for informed decision-making and policy development. By synthesizing research findings and staying abreast of emerging trends, stakeholders can navigate the complex landscape of *Cannabis sativa* use with a focus on promoting public health and well-being.

### 3.2. Erythroxylum coca (Coca Plant)

#### 3.2.1. Historical Background

*Erythroxylum coca*, commonly known as the coca plant, has a long history of traditional use by indigenous South American tribes for various purposes, including stimulant, anesthetic, religious, and recreational uses [113]. Belonging to the Erythroxylaceae family, this plant has been cultivated for thousands of years, with evidence of its use dating back at least 8000 years ago [114]. The plant has been cultivated for its leaves, which contain the alkaloid cocaine, a potent stimulant and narcotic substance [115]. The coca plant was one of the first domesticated plant species, providing nutritional, medicinal, and digestive properties to ancient civilizations through the practice of chewing its leaves [116]. The use of coca for medicinal purposes, particularly in treating altitude sickness, has been a centuries-old tradition among the native peoples of South America [115].

#### 3.2.2. Drug Policy

Erythroxylum species, especially *Erythroxylum coca*, are globally banned for cultivation and consumption. Current legislation only allows their use for medical and scientific purposes or as an extract in drinks such as Coca-Cola. However, in Bolivia, Colombia, and Peru, cocaine-based products such as medical products and food are available on the market, although coca continues to be sold illegally, against the law [56].

#### 3.2.3. Pharmacological Properties and Health Implications

Cocaine, the primary alkaloid found in *Erythroxylum coca*, has been the subject of extensive research due to its pharmacological effects and societal implications. The alkaloid is naturally found in the leaves of coca plants. It has been associated with anesthetic properties, with cocaine being a prototype for modern local anesthetics [116]. Studies have shown that cocaine and cinnamoyl-cocaine in *Erythroxylum coca* are stored in the vacuoles of the plants and complexed with hydroxycinnamoyl quinate esters [57]. Additionally, research has indicated that cocaine functions as a plant’s natural insecticide, suggesting its role in defense mechanisms against herbivores [117].

The domestication and cultivation of *Erythroxylum coca* have been of interest to researchers studying the origins and genetic diversity of the plant. Genomic studies have revealed multiple independent domestications of coca from its progenitor, *Erythroxylum gracilipes*, highlighting the evolutionary history of the plant. Phylogenetic analyses have provided insights into the relationships within the Erythroxylum genus, informing taxonomy, biogeography, and the domestication of coca species [118]. Furthermore, the complete genome sequences of *Erythroxylum coca* and *Erythroxylum novogranatense* have been elucidated, shedding light on the genetic makeup of these important cultigens [119].

Chemical analyses of *Erythroxylum coca* have identified various alkaloids present in the plant, including cocaine, cinnamoyl-cocaine, and tropane alkaloids [115]. The biosynthesis of tropane alkaloids in *Erythroxylum coca* has been investigated, revealing the enzymatic pathways involved in the production of these pharmacologically significant compounds [119]. Studies have also assessed the distribution of cocaine in different wild Erythroxylum species, highlighting variations in alkaloid content among different plant populations [120].

In contemporary times, the consumption of coca leaves and derived products has evolved, with a shift toward recreational and narcotic purposes. The chemical compounds found in *Erythroxylum coca*, such as tropane alkaloids like cocaine, are responsible for its psychoactive effects [121]. These compounds act on the central nervous system, leading to stimulant effects that induce euphoria and increased energy levels, making them desirable for recreational use. However, the consumption of coca products, especially in the form of cocaine, is associated with various adverse effects on health [122].

The narcotic and recreational effects of coca products, particularly cocaine, are well documented. Cocaine acts as a potent stimulant, affecting neurotransmitter levels in the brain, particularly dopamine, which leads to its addictive properties and potential for abuse [123]. Chronic use of cocaine can result in a range of adverse health effects, including cardiovascular complications, neurological disorders, psychiatric symptoms, and even overdose-related fatalities. The addictive nature of cocaine further exacerbates its negative impact on individuals, leading to a cycle of dependence and potential long-term health consequences [121].

#### 3.2.4. Mechanism of Action

The primary anesthetic effect of cocaine is due to the inhibition of voltage-gated sodium channels, blocking the entry of sodium ions into cells and, consequently, preventing the transmission of nerve impulses. Since the transmission of nerve impulses is blocked when sodium ions are inhibited, the use of cocaine as a topical anesthetic is becoming less common. At the dopaminergic level, cocaine inhibits the dopamine transporter (DAT), causing the accumulation of extracellular dopamine, which produces euphoria. At the cardiac level, cocaine exhibits cardiotoxic effects by inhibiting norepinephrine reuptake, leading to vasoconstriction. In the central nervous system, by interacting with sigma and kappa-opioid receptors, cocaine affects the structure and function of dopamine receptors, thus contributing to its psychoactive effects [35].

From a public health perspective, the widespread use of coca products, especially cocaine, poses significant challenges. The addictive nature of cocaine contributes to substance abuse disorders and societal issues related to drug dependence [124]. Additionally, the illicit production and trafficking of cocaine have fueled organized crime and violence in regions where coca cultivation is prevalent, further complicating efforts to address the public health implications of coca consumption. The adverse health outcomes associated with cocaine use underscore the importance of comprehensive strategies to prevent substance abuse and provide support for individuals struggling with addiction [125].

#### 3.2.5. Future Perspectives on Public Health

Looking toward the future, addressing the public health implications of coca consumption requires a multifaceted approach. Education and awareness programs are essential to inform the public about the risks associated with coca products, particularly cocaine, and to promote healthy lifestyle choices. Additionally, regulatory measures and law enforcement efforts are crucial to combat illicit drug production and trafficking, reducing the availability of coca products in the black market. Treatment and rehabilitation services play a vital role in supporting individuals affected by substance abuse disorders, offering them the necessary resources to overcome addiction and improve their overall well-being [126].

#### 3.2.6. General Conclusion

Overall, *Erythroxylum coca*, with its historical significance and psychoactive properties, has been a subject of interest for recreational and narcotic purposes. The chemical compounds present in coca, such as cocaine, have potent effects on the central nervous system, leading to both recreational enjoyment and severe health consequences. Addressing the adverse effects of coca consumption, particularly cocaine abuse, is paramount for public health. By employing multifaceted approaches integrating education, regulation, enforcement, and treatment, societies can effectively address the detrimental effects of coca products on individuals and communities. Extending from historical indigenous practices to modern-day public health challenges, additional research is imperative to elucidate this culturally and scientifically noteworthy botanical specimen’s historical, current, and prospective ramifications.

### 3.3. Papaver somniferum (Opium Poppy)

#### 3.3.1. Historical Background

*Papaver somniferum*, commonly known as opium poppy, is an annual plant belonging to the Papaveraceae family with a rich historical significance as an ancient medicinal plant. It is renowned for being the primary commercial source of various narcotic analgesics such as morphine, codeine, and their semi-synthetic derivatives like oxycodone and hydrocodone. The plant’s significance dates back to ancient times when it was utilized for its medicinal properties and continues to be the primary commercial origin of these potent analgesics [127]. Morphine and codeine, the primary alkaloids derived from *Papaver somniferum*, are known for their powerful narcotic effects and are widely used in the pharmaceutical industry [128].

#### 3.3.2. Drug Policy

The cultivation of opium is only possible in two situations well regulated by law, namely for medicinal purposes and resin. For example, in India, poppy cultivation is legalized under the Narcotic Drugs and Psychotropic Substances (NDPS) Act for medicinal purposes only, under government supervision. Every year, a representative from the government is responsible for verifying the requirements (land area, patience, quality standards), which, if farmers do not fulfill, they do not get approval to cultivate. To ensure control and to reduce the risk of abuse, Indian farmers are obliged to sell their produce to the government at prices set by the authorities [129].

#### 3.3.3. Pharmacological Properties and Health Implications

Contemporary consumption trends of *Papaver somniferum* highlight its continued relevance in the production of opiates and opioids for medical and recreational purposes [130]. The plant’s alkaloids, particularly morphine and codeine, are central to its pharmacological importance and are responsible for its narcotic effects. The biosynthesis of these alkaloids occurs in specialized cells within the plant, known as laticifers, emphasizing the intricate biochemical processes involved in producing these potent [131]. The presence of these alkaloids in *Papaver somniferum* has led to its widespread cultivation and utilization for both medicinal and recreational purposes [130].

The chemical compounds responsible for the narcotic and recreational effects of *Papaver somniferum* are primarily benzylisoquinoline alkaloids, including morphine, codeine, and noscapine [130]. These alkaloids play a crucial role in the plant’s pharmacological properties, with morphine being a key component due to its potent analgesic effects [131]. The biosynthesis of these alkaloids involves complex pathways and enzymatic reactions within the plant, highlighting the sophisticated mechanisms underlying the production of these bioactive compounds [132]. The presence of these alkaloids in opium poppy seeds has raised concerns regarding consumer exposure to these potent substances [133].

In terms of adverse effects, the consumption of *Papaver somniferum* and its derivatives for narcotic and recreational purposes can lead to various health risks and unfavorable outcomes. Opioids derived from the plant, such as morphine and codeine, are known for their addictive properties and potential for substance abuse. Prolonged use of these substances can result in physical dependence, tolerance, and withdrawal symptoms, contributing to the global opioid crisis. Additionally, the misuse of opium poppy products can lead to overdose, respiratory depression, and even death, underscoring the severe consequences associated with the recreational and non-medical use of these substances [134].

#### 3.3.4. Mechanism of Action

Papaverine is a potent vasodilator that increases levels of cAMP and cGMP by inhibiting the phosphodiesterases that break them down. These intracellular messengers are responsible for smooth muscle relaxation. Papaverine may also inhibit the release of intracellular calcium ions, thereby reducing muscle contractions and blocking the entry of ions into the cells, decreasing contraction impulses [135].

#### 3.3.5. Future Perspectives on Public Health

Looking toward future implications for public health, addressing the challenges posed by the widespread availability and misuse of *Papaver somniferum*-derived products is essential. Strategies focusing on harm reduction, addiction treatment, and public awareness campaigns are crucial in mitigating the negative impact of opioid abuse on individuals and communities [136]. Furthermore, research efforts aimed at developing alternative pain management strategies that reduce reliance on opioid medications derived from opium poppy can help alleviate the burden of opioid-related harm [137]. By promoting responsible prescribing practices, enhancing addiction treatment services, and implementing comprehensive public health interventions, the adverse effects of *Papaver somniferum* consumption can be effectively managed to safeguard public health and well-being.

#### 3.3.6. General Conclusion

Overall, *Papaver somniferum* is a plant of immense significance, with a long history of medicinal use and a complex relationship with human society. Its chemical compounds, particularly the benzylisoquinoline alkaloids, are responsible for its therapeutic and recreational effects, making it a subject of interest for researchers and policymakers alike. While the plant offers valuable compounds for pharmaceutical purposes, the potential adverse impacts of its recreational use necessitate careful regulation and public health interventions. Future research on *Papaver somniferum* holds promise for uncovering new bioactive compounds and understanding its genetic diversity, paving the way for advancements in medicine and plant science.

### 3.4. Mitragyna speciosa (Kratom)

#### 3.4.1. Historical Background

*Mitragyna speciosa*, commonly known as kratom, is a tropical tree belonging to the Rubiaceae family, historically used in traditional medicine practices in regions of Africa and Southeast Asia [85]. While traditionally used for its analgesic properties mediated by opioid receptors, it has gained popularity as a recreational psychoactive drug in Western countries [137,138]. Additionally, it has been reported to have psychoactive effects and opium-like characteristics. It has been used to manage opioid addiction and withdrawal symptoms [139]. The plant has a long history of use in Southeast Asia for its stimulant effects, as a traditional herbal medicine, and as a substitute for opium and alcohol. [138]. *Mitragyna speciosa* has also been employed to alleviate pain, hypertension, cough, and diarrhea and as a replacement for morphine in treating people with an addiction [140]. Overall, the traditional uses of *Mitragyna speciosa* encompass many medicinal and psychoactive applications that have been recognized for centuries.

#### 3.4.2. Drug Policy

Until now, the legalization of the use of kratom, classified in 2016 by the Drug Enforcement Administration (DEA) as a Category I substance, has not yet been regulated worldwide due to a total lack of safety. In Australia, kratom is also not used for medical purposes. However, in Thailand and most states in America, because there are no cases of violence or overdose following its consumption, the use of kratom for medical purposes is possible. In America, kratom is on the market only as a dietary supplement and as a food, and until now, there has been no regulation to ensure its safe use in medical treatment [141].

#### 3.4.3. Pharmacological Properties and Health Implications

The pharmacology of *Mitragyna speciosa* is complex, with alkaloids such as mitragynine binding to opioid receptors and influencing pain perception [142]. Furthermore, the directed biosynthesis of mitragynine stereoisomers demonstrates the potential for manipulating the chemical composition of kratom for specific purposes [143]. In addition, *Mitragyna speciosa* leaves contain other alkaloids, such as 7-hydroxy mitragynine, which are identified as the main psychoactive components responsible for its effects [93].

#### 3.4.4. Mechanism of Action

Kratom is a plant recognized for its numerous therapeutic effects, such as analgesic, antipyretic, sedative, antimicrobial, anti-inflammatory, and dopaminergic stimulant. Its natural alkaloid, mitragynine, is responsible for these effects, which induce specific narcotic effects through its interaction with numerous receptors in the nervous system. At low doses, mitragynine exhibits stimulant effects, while at high doses, its agonist action on opioid I receptors and antagonist action on d receptors produces effects specific to conventional opioids. Mitragynine inhibits the COX-2 enzyme and prostaglandin E2 to exert its anti-inflammatory effect. For its analgesic effect, it activates a-2 adrenergic receptors and inhibits Ca^2+^ channels [144,145].

In the United States, there has been an increase in the use of *Mitragyna speciosa* for self-treatment of pain and opioid addiction [146]. While the plant shows promise for pain management and opioid withdrawal, chronic use has been associated with punishment resistance in natural reward-seeking behavior and impaired learning in animal studies [147]. Concerns have also been raised regarding the potential adverse effects, such as addiction and psychomotor impairment associated with the consumption of botanical preparations of *Mitragyna speciosa* to alleviate negative emotions [148,149].

#### 3.4.5. Future Perspectives on Public Health

Looking toward future public health implications, *Mitragyna speciosa* shows promising potential due to its anti-inflammatory effects and therapeutic properties [150]. Moreover, its cellular toxicology and the risks associated with its dominant alkaloid, mitragynine, are being explored [151]. Understanding the effects of *Mitragyna speciosa* on the central nervous system and behavior is crucial as research progresses [152]. Additionally, exploring this plant’s genetic diversity and metabolomics can offer valuable insights into its variability and chemical composition [143,153].

#### 3.4.6. General Conclusion

Overall, *Mitragyna speciosa* presents a nuanced landscape for recreational and narcotic purposes, with the potential for pain management and opioid addiction treatment. However, the negative consequences of its consumption and associated health risks necessitate careful consideration. A balanced approach that explores its therapeutic potential while mitigating adverse effects is essential for shaping future public health policies and interventions as research on *Mitragyna speciosa* advances.

### 3.5. Catha edulis (Khat)

#### 3.5.1. Historical Background

*Catha edulis*, commonly known as khat, is a plant with historical and cultural significance, particularly in regions like East Africa and the Arabian Peninsula. Belonging to the Celastraceae family, khat has been traditionally chewed for its stimulant effects, akin to amphetamines, due to the presence of cathinone, a beta-ketone amphetamine analog found in the leaves of the plant [154]. The contemporary consumption trends of khat involve its use for recreational purposes, inducing a sense of euphoria and increased energy levels, making it popular in social settings. This plant has psychoactive effects and is widely used globally, with a prevalence of chewing fresh leaves and twigs for its stimulating properties [155].

#### 3.5.2. Drug Policy

Until the 1970s, khat was not considered a hazardous substance, as the decision of the United Nations Commission on Narcotic Drugs allowed each country to decide how it wished to regulate its use. However, in 1988, following the discovery of cathinone—the active substance in khat most likely to cause the most adverse effects in the general population—the WHO included it in Category I of psychoactive substances. In recent years, the ECDD has considered that khat does not pose a very high risk of abuse and addiction, so countries such as Kenya and Ethiopia have legalized the use of khat. However, there are also countries where possession and consumption are illegal, such as Italy, Germany, Saudi Arabia, and Sweden [156].

#### 3.5.3. Pharmacological Properties and Health Implications

The chemical compounds responsible for khat’s narcotic and recreational effects include alkaloids, tannins, flavonoids, terpenes, sterols, and essential oils found in its leaves. These compounds contribute to the plant’s psychoactive properties, making it sought after for its stimulant effects [157].

However, alongside its recreational use, khat consumption has been associated with various adverse effects on health. Studies have reported cases of acute liver failure and autoimmune hepatitis among consumers of *Catha edulis*, highlighting potential hepatotoxicity induced by its use [158]. Additionally, research has shown that khat consumption can lead to adverse effects such as cardiovascular issues and other internal medical problems, emphasizing the need for further investigation into its health implications [159]. Furthermore, the long-term toxicological effects of khat leaves have been studied in animals, revealing increased levels of enzymes like alkaline phosphatase and alanine aminotransferase, indicating potential liver damage [160]. These findings underscore the importance of understanding the risks associated with khat consumption and its impact on various physiological systems.

#### 3.5.4. Mechanism of Action

Cathinone, the main active compound in khat, primarily acts at the nervous system level by inhibiting dopamine-specific transporters (DAT) and serotonin-specific transporters (SERT). At the dopaminergic level, cathinone has a slightly lower capacity than amphetamine to release tritiated dopamine (3H-DA). However, because it inhibits catecholamine transporters, much like methamphetamine, it is considered similar to it [161].

#### 3.5.5. Future Perspectives on Public Health

Looking toward the future implications for public health, addressing the growing concerns surrounding khat use and its associated health risks is crucial. Studies have highlighted the need for further research into the pharmacological and medical aspects of khat, considering its widespread social use and potential for substance-related disorders [162]. Moreover, investigations into the development of synthetic cathinone, derived from khat, as substances of abuse have raised concerns about the toxicology and classification of these compounds, necessitating regulatory measures to mitigate their harmful effects. The emergence of designer drugs based on cathinone derivatives underscores the importance of monitoring and regulating the use of such substances to safeguard public health [163].

#### 3.5.6. General Conclusion

Overall, *Catha edulis* holds a complex position in society, being valued by consumers for its stimulant properties while also posing significant health risks. Its historical significance, contemporary consumption trends, chemical compounds responsible for its effects, negative health implications, and future implications for public health all contribute to a nuanced understanding of this plant. As further research sheds light on the pharmacological, toxicological, and societal aspects of khat use, it is essential to balance cultural practices with public health considerations to ensure the well-being of individuals in communities where khat consumption is prevalent.

### 3.6. Salvia divinorum (Holy Sage)

#### 3.6.1. Historical Background

*Salvia divinorum*, commonly known as holy sage, is a perennial plant from the Lamiaceae family, native to Mexico, particularly Oaxaca. It is distinguished among Salvia species by containing salvinorin A, a potent psychoactive compound responsible for its hallucinogenic properties [164]. Initially used by the Mazatec people for spiritual and medicinal purposes, *Salvia divinorum* has gained popularity globally for recreational use, often as a substitute for marijuana [165].

#### 3.6.2. Drug Policy

In the United States, to date, according to the Drug Enforcement Administration (DEA), possession and distribution of *Salvia divinorum* is not prohibited. However, a few countries, such as Australia, Italy, Denmark, and South Korea, have in recent years passed laws banning possession of the plant, classifying it as a Class I substance with a high potential for abuse and no accepted medical use [166].

#### 3.6.3. Pharmacological Properties and Health Implications

Salvinorin A, the primary active ingredient in *Salvia divinorum*, acts as a highly selective kappa-opioid receptor agonist, leading to hallucinogenic effects such as visual distortions and synesthesia [167].

Studies on the pharmacokinetics and pharmacodynamics of salvinorin A have provided insights into its absorption, distribution, metabolism, and excretion in the human body [168]. Salvinorin A’s impact extends beyond the central nervous system, affecting colonic transit and neurogenic ion transport, suggesting a role in gastrointestinal motility regulation [169]. Furthermore, its inhibitory effects on enteric cholinergic transmission point to potential therapeutic applications in gastrointestinal [165]. Despite its intriguing pharmacological profile, the recreational use of *Salvia divinorum* poses risks. Documented effects of salvinorin A in humans include transient but intense alterations in perception and cognition [170]. Concerns about its abuse potential, impact on the central nervous system, and behavioral effects highlight the importance of caution in its consumption [171]. The availability of *Salvia divinorum* through online sources has raised concerns about misuse among adolescents and associated toxicological implications [172].

#### 3.6.4. Mechanism of Action

Salvinorin A is responsible for the mechanism of action. This compound binds to the amino acids of the kappa-opioid receptor (KOP) through hydrophobic bonds, leading to analgesia. Salvinorin A is a selective agonist of the KOP, and its activation produces sedation and euphoria by increasing norepinephrine levels while inhibiting dopamine and serotonin. Additionally, salvinorin A interacts with the dopamine transporter (DAT) via KOP, forming heterodimers that interact with cannabinoid CB1 receptors [173].

#### 3.6.5. Future Perspectives on Public Health

Understanding the effects of *Salvia divinorum* is crucial for public health. Research focusing on the molecular mechanisms of salvinorin A, its long-term impact on cognitive function and mental health, and the development of safer alternatives for pain management can inform regulatory measures and harm reduction strategies [174,175].

#### 3.6.6. General Conclusion

Overall, *Salvia divinorum*, with its hallmark compound salvinorin A, holds a concerning place among recreational substances. Its historical significance, contemporary use patterns, and pharmacological properties require a comprehensive approach to address its consumption, considering its benefits and risks. A balanced perspective can guide further scientific inquiry and societal well-being by exploring its chemical compounds, mechanisms of action, negative consequences, and future public health implications.

### 3.7. Lophophora williamsii (Peyote)

#### 3.7.1. Historical Background

*Lophophora williamsii*, commonly known as peyote, is a small, spineless cactus belonging to the Cactaceae family [176]. This cactus is primarily found in dry regions from Central Mexico to Texas, particularly along the Rio Grande [177]. It holds significant historical and cultural importance, especially among indigenous peoples of North America. The plant contains various biologically active alkaloids, with mescaline being the most well-known and studied compound [178]. Mescaline, a phenethylamine compound, is the primary psychoactive component responsible for the narcotic and recreational effects associated with peyote consumption [179]. Despite its long history of use in spiritual and healing rituals, the exact mechanisms through which mescaline exerts its psychoactive effects remain incompletely understood [180].

#### 3.7.2. Drug Policy

Peyote is not fully legalized, with restrictions on its use still in place. Soldiers in Vietnam were using peyote in the war at the same time as the number of drug-related deaths was rising worldwide. Since the 1970s, peyote has been considered a high-risk plant for health, being classified as a category one substance with a high risk of abuse. However, in 1994, following protests by Indigenous Americans, its use was regulated for religious purposes only [181].

#### 3.7.3. Pharmacological Properties and Health Implications

Peyote has been traditionally used for millennia in spiritual and folk healing rituals, reflecting its deep-rooted historical significance [182]. The plant’s psychoactive properties have intrigued researchers, leading to investigations into its chemical composition and structure–activity relationships [183]. Studies have highlighted the presence of mescaline in high concentrations in *Lophophora williamsii*, emphasizing its role as a potent hallucinogen [184]. Additionally, the identification of peyote based on morphological differences, mescaline content, and genetic sequences has furthered our understanding of this plant and its distinct species [185].

The consumption trends surrounding *Lophophora williamsii* have evolved, with the plant being recognized for its psychoactive properties and potential therapeutic benefits. The chemical compounds found in *Lophophora williamsii*, particularly mescaline, play a pivotal role in inducing hallucinogenic effects that alter perception, time sense, and visual phenomena upon consumption [186,187]. While peyote has been historically used in cultural and religious contexts, contemporary interest in its recreational use has raised concerns about its negative effects on health [152]. The consumption of *Lophophora williamsii* provides feelings of euphoria and relaxation, which may appeal to individuals seeking relief from stress and negative emotions. Its reported ability to enhance creativity and introspection has made it popular among artists and spiritual seekers. However, it is important to note that repeated use of peyote can lead to psychological dependence, especially in individuals who use it to escape reality or cope with emotional distress [180,184,188].

#### 3.7.4. Mechanism of Action

Mescaline, the primary active alkaloid in peyote, acts as an agonist of 5-HT2 receptors in the brain and blood vessels. By activating these receptors, mescaline stimulates phospholipase C via Gq proteins, generating chemical signals and releasing Ca²⁺ ions from the endoplasmic reticulum. In addition, due to its interaction with neuronal serotonergic processes, mescaline may, depending on dose, reduce or increase levels of 5-HIAA, the primary metabolite of serotonin [179].

#### 3.7.5. Future Perspectives on Public Health

Despite its cultural significance and potential therapeutic properties, the overharvesting and habitat loss of *Lophophora williamsii* pose significant threats to its survival in the wild [189]. As researchers continue to explore the pharmacological and toxicological aspects of peyote, there is a growing need to address the implications of its recreational use on public health. Understanding the mechanisms of action of mescaline and other alkaloids present in *Lophophora williamsii* is crucial for developing harm reduction strategies and promoting safe consumption practices [190].

#### 3.7.6. General Conclusion

Overall, *Lophophora williamsii*, specifically peyote, stands out as a plant with rich historical significance, complex chemical composition, and diverse consumption trends. While its psychoactive properties have captivated researchers and enthusiasts alike, the need to balance cultural traditions with public health concerns remains paramount. Further research into the pharmacology, chemistry, and conservation of *Lophophora williamsii* is essential to ensure its sustainable use and preservation for future generations.

### 3.8. Banisteriopsis caapi (Soul Vine)

#### 3.8.1. Historical Background

*Banisteriopsis caapi*, a plant native to the Amazon basin, is historically and currently significant for its use in recreational and narcotic contexts. Belonging to the Malpighiaceae family, this plant is a key ingredient in the preparation of the psychoactive brew known as ayahuasca, which is traditionally used in religious ceremonies and is increasingly popular globally [191]. The psychoactive effects of ayahuasca are attributed to the combination of *Banisteriopsis caapi*, containing β-carboline alkaloids like harmine, harmaline, and tetrahydroharmine, and *Psychotria viridis*, which provides the hallucinogenic compound *N*,*N*-dimethyltryptamine (DMT) [192].

#### 3.8.2. Drug Policy

The regulations surrounding ayahuasca legislation are highly controversial, requiring close monitoring, although the primary active substance, DMT, was classified in 1971 as a Category I drug. Despite its addictive potential, in 2006, the Religious Freedom Restoration Act (RFRA) was passed, which states that ayahuasca can only be used for religious ceremonial purposes in America and Canada [193].

#### 3.8.3. Pharmacological Properties and Health Implications

Understanding the chemical composition of *Banisteriopsis caapi* is crucial to understanding its effects. The plant contains β-carbolines, such as harmine and harmaline, which act as potent monoamine oxidase inhibitors (MAOIs) [194]. These compounds are essential in the pharmacological actions of ayahuasca, with harmine specifically inhibiting protein kinase DYRK1A and interfering with neurite formation, indicating its impact on neural processes [195]. Additionally, *Banisteriopsis caapi* has been found to stimulate adult neurogenesis in vitro, suggesting potential neuroprotective and regenerative properties [196].

The consumption trends of *Banisteriopsis caapi* and ayahuasca have gained popularity in both traditional and contemporary contexts. The plant’s alkaloids, particularly the β-carbolines, inhibit monoamine oxidase and activate the 5-HT 2A receptor, leading to hallucinatory experiences in users [197]. Furthermore, *Banisteriopsis caapi* has been researched for potential therapeutic applications in neurodegenerative disorders like Parkinson’s disease, with its chemical profile showing promise in treating such conditions [198].

Despite its potential benefits, the excessive consumption of *Banisteriopsis caapi* and ayahuasca is not without negative effects. Therefore, caution should be exercised regarding the long-term and recreational use of *Banisteriopsis caapi* due to its potential adverse effects on cardiovascular health and cognitive functions [192]. It is essential to note that the plant’s alkaloids can have intense cardiovascular effects when used intravenously in large doses [199]. Additionally, the synergistic interaction of β-carbolines and *N*, *N*-dimethyltryptamine in *Banisteriopsis caapi* may impact cognitive thinking styles and affect, potentially leading to ego dissolution [200,201]. Moreover, the β-carboline alkaloids present in *Banisteriopsis caapi* have been associated with mutagenicity, raising concerns about the safety of prolonged or excessive consumption [202].

#### 3.8.4. Future Perspectives on Public Health

Looking ahead, the future implications for public health regarding the recreational and narcotic use of *Banisteriopsis caapi* are multifaceted. While research indicates the therapeutic potential of this plant in treating neurological disorders, comprehensive studies on its long-term effects and safety profile are needed [203]. Public health initiatives should focus on promoting responsible use, conducting further research on its pharmacological properties, and raising awareness about the potential risks associated with its consumption.

#### 3.8.5. General Conclusion

Overall, *Banisteriopsis caapi* is a plant of great significance due to its historical use in traditional rituals and its contemporary relevance in recreational and narcotic contexts. The chemical compounds in this plant, particularly the β-carbolines, play a crucial role in its psychoactive effects and potential therapeutic applications. However, the adverse effects and safety concerns associated with its consumption highlight the importance of continued research and public health measures to ensure the safe and informed use of *Banisteriopsis caapi*.

### 3.9. Datura stramonium (Devil’s Trumpet)

#### 3.9.1. Historical Background

*Datura stramonium*, commonly known as Jimson weed or devil’s trumpet, belongs to the Solanaceae family and is widely distributed across regions like Asia, the United States, Canada, and the West Indies [204]. This plant has a rich historical significance, with references to its use dating back centuries for various purposes. In contemporary times, *Datura stramonium* has gained popularity for its recreational and narcotic effects, leading to concerning consumption trends. The plant contains various chemical compounds responsible for its effects, including alkaloids, saponins, tannins, flavonoids, and glycosides. These compounds contribute to the plant’s pharmacological properties, making it attractive for recreational use [205].

#### 3.9.2. Drug Policy

Due to limited safety and efficacy data, the Food and Drug Administration (FDA) does not consider the belladonna alkaloids present in *Datura stramonium* safe or effective in over-the-counter cough and cold preparations. In this regard, several states, including Tennessee, Connecticut, and New Jersey, have passed legislation to control the use of jimsonweed. In Tennessee, possessing jimsonweed seeds on school grounds is classified as a Class A misdemeanor. Connecticut includes *Datura stramonium* in the category of “restricted drugs or substances”. In New Jersey, the use or possession of stramonium preparations is illegal unless prescribed by a licensed physician. However, at the federal level, Jimsonweed (*Datura stramonium*) is not regulated as an illegal substance [206].

#### 3.9.3. Pharmacological Properties and Health Implications

The mechanism behind the narcotic and recreational purposes of *Datura stramonium* lies in its alkaloid content, particularly hyoscyamine, scopolamine, and atropine. These alkaloids have potent psychoactive effects on the central nervous system, leading to visual hallucinations, agitation, lack of coordination, aggressiveness, delirium, drowsiness leading to comatose states, and other altered states of consciousness when consumed in sufficient quantities. The presence of these compounds makes *Datura stramonium* a sought-after plant for individuals seeking intense psychoactive experiences [207,208]. It is sometimes combined with tobacco to create a potent mixture for inhalation or smoking, resulting in psychoactive effects that can last for several days. These effects can pose potential dangers compared to other hallucinogens like LSD or psilocybin [209].

However, the consumption of *Datura stramonium* for recreational or narcotic purposes is not without its risks. The plant is known to cause a range of adverse effects, including anticholinergic toxicity, which can manifest as dry mouth, blurred vision, tachycardia, urinary retention, and central nervous system disturbances [210]. In severe cases, *Datura stramonium* poisoning can lead to coma, seizures, and even death, highlighting the severe consequences of its misuse [211]. Additionally, *Datura stramonium* has been implicated in cases of acute renal failure, further underscoring the potential dangers associated with its consumption [212].

Consumption of Datura seeds for their hallucinogenic properties has a long history, with Native Americans using them as a euphoric [213]. Datura plants contain tropane alkaloids, particularly in their seeds, flowers, leaves, and roots, responsible for their hallucinogenic effects [211]. These alkaloids can lead to severe anticholinergic effects, causing toxicity when any part of the plant is consumed [212]. The intentional ingestion of Datura for its psychoactive effects has increased, especially among adolescents and youths [213,214]. The toxicity of Datura largely depends on the solvent used for extraction, with the polarity and non-polarity of the solvent being linked to the plant’s toxicity [215]. Furthermore, Datura could be consumed in various ways, including ingesting the seeds, flowers, or roots, which could lead to adverse physiological changes and severe anticholinergic reactions [205,216]. The plant’s hallucinogenic properties have led to its use for substance abuse, with ingestion, usually of the seeds, being the most common route of exposure [217]. Additionally, Datura producers have been known to create hallucination-inducing brews by boiling leaves and seeds, sometimes mixing Datura with other substances [218].

The consumption of *Datura stramonium* seeds for hallucinogenic effects and other mind-altering effects is a practice that poses significant risks and dangers to individuals [219]. Long-term consumption of Datura seeds has been associated with severe consequences, including the onset of paranoid schizophrenia, aggressive behavior, and altered states of consciousness [93]. Additionally, *Datura stramonium* seed extracts have been found to exhibit toxicity affecting various organs such as the liver, kidney, heart, and brain, with mechanisms including lipid peroxidation contributing to oxidative stress in these organs [215]. Furthermore, acute intoxication with *Datura stramonium* seeds can lead to symptoms such as coma, further highlighting the dangerous effects of this plant [210].

#### 3.9.4. Mechanism of Action

Hyoscyamine, scopolamine, and atropine, the alkaloids found in D. stramonium, act on the nervous system by antagonizing the muscarinic receptors M2, M4, and M5 of acetylcholine. In the context of asthma, blocking these receptors induces bronchodilation. Additionally, these alkaloids can be used in the treatment of gliomas, as they can induce cell apoptosis and neutralize free radicals, thereby preventing oxidative stress [220,221].

#### 3.9.5. Future Perspectives on Public Health

Looking toward the future, the implications of *Datura stramonium* use for public health are significant. As the plant continues to be consumed for recreational purposes, there is a growing need for public health interventions to raise awareness about its dangers and regulate it [204]. Education campaigns targeting at-risk populations, such as adolescents and young adults, could help prevent instances of *Datura stramonium* poisoning and associated health complications. Furthermore, healthcare providers should be trained to recognize the signs of Datura intoxication and provide appropriate medical care to affected individuals [222].

#### 3.9.6. General Conclusion

Overall, *Datura stramonium* holds a complex position in society, with its historical significance, contemporary consumption trends, pharmacological mechanisms, chemical compounds, adverse effects, and public health implications all contributing to a nuanced understanding of this plant. While it may offer psychoactive experiences sought by some, the risks associated with its use cannot be overlooked. A balanced approach considering *Datura stramonium*’s cultural significance and potential harms is essential for promoting public health and safety.

### 3.10. Ipomoea violacea, Ipomoea tricolor (Morning Glory)

#### 3.10.1. Historical Background

Ipomoea, a genus of flowering plants in the family Convolvulaceae, commonly known as morning glories, has a rich historical significance dating back to its traditional uses in various cultures for medicinal, recreational, and narcotic purposes. The genus includes several species like *Ipomoea violacea*, *Ipomoea tricolor*, and *Ipomoea carnea*. In contemporary times, Ipomoea species have gained attention for their psychoactive effects, with some being used as “herbal highs” due to their active principles. These plants are often easily accessible through online platforms, contributing to their widespread consumption [93].

#### 3.10.2. Drug Policy

Although the authorities have made numerous efforts to stop the cultivation, possession, and use of drugs, the number of people and cases of drug users and deaths associated with them is still on the rise. Nigerian communities are using psychoactive plants such as Morning Glory for non-medical purposes, particularly in religious ceremonies. The state has legalized their use for recreational purposes only based on their low safety [221].

#### 3.10.3. Pharmacological Properties and Health Implications

One of the key chemical compounds found in Ipomoea species that are responsible for their effects is resin glycosides such as tricolorin A [223]. The plants also contain various chemical compounds that contribute to their biological activities, such as flavonoids and phenols [224,225]. Additionally, various phytochemical constituents present in Ipomoea species have been associated with anti-inflammatory, detoxification, and therapeutic activities, showcasing their potential in treating various diseases such as diabetes, hypertension, and inflammation. These diverse pharmacological activities highlight the versatility of Ipomoea plants in traditional medicine and alternative therapies [226]. The presence of compounds like tetrasaccharides and pentasaccharide glycosides in Ipomoea roots has been linked to antimycobacterial activity, cytotoxicity, and effects on the central nervous system, indicating a complex interplay of bioactive components within these plants [227].

While Ipomoea species offer a range of potential benefits, there are also adverse effects associated with their consumption. Studies have highlighted potential toxicity risks, especially when wild Ipomoea varieties are ingested, emphasizing the importance of properly identifying and understanding these plants to prevent adverse outcomes [228]. Furthermore, compounds like tyrianthinic acids in certain Ipomoea species raise concerns about cytotoxicity and central nervous system effects, underscoring the need for cautious consumption [227].

#### 3.10.4. Mechanism of Action

The hallucinogenic effects of Ipomoea are attributed to the presence of the ergine alkaloid d-lysergic acid amide (LSA). LSA is known to produce hallucinogenic and psychedelic effects when ingested [229]. These effects are similar to those induced by other hallucinogens like LSD, psilocybin, and mescaline, which profoundly impact perception, cognition, and mood [230]. The psychoactive properties of LSA are distinct from LSD and more akin to scopolamine [231]. LSD has a complex mechanism of action, which is responsible for a wide range of effects. At the serotonergic level, it acts both on 5-HT1 receptors (5-HT1A, 5-HT1B, 5-HT1D), with an inhibitory effect, and on 5-HT2 receptors as a stimulator, being able to modify its effect. Through its agonist effect on 5-HT2A receptors, LSD is thus considered to be responsible for the hallucinogenic effects following administration of Ipomoea, but also of other substances in the class of phenethylamine and indolamine derivatives. At the dopaminergic level, LSD interacts with D1 and D2 receptors about 90 min after administration, while activation of serotonergic receptors occurs after about 30 min [232].

Moreover, the alkaloids present in Ipomoea, such as ergine and ergometrine, are known to have psychoactive properties that contribute to the hallucinogenic experiences associated with consuming Ipomoea [233].

#### 3.10.5. Future Perspectives on Public Health

Looking ahead, the future implications for public health concerning Ipomoea plants are multifaceted. On the one hand, the rich chemical diversity in these species offers opportunities for drug discovery and the development of novel therapeutic agents for various [234]. However, the recreational and narcotic potential of certain Ipomoea species also pose challenges in terms of regulation, safety, and public awareness regarding their effects. As more research delves into Ipomoea plants’ pharmacological, toxicological, and ecological aspects, a comprehensive understanding of their benefits and risks will be crucial for informed decision-making in healthcare and regulatory frameworks [233].

#### 3.10.6. General Conclusion

Overall, Ipomoea plants stand at the intersection of traditional medicine, recreational use, and scientific exploration, offering a complex tapestry of bioactive compounds with diverse effects on the human body. From their historical significance to contemporary consumption trends and future implications for public health, Ipomoea species continue to intrigue researchers and health professionals alike. By unraveling the mechanisms behind their narcotic and recreational properties, identifying key chemical compounds responsible for these effects, understanding the negative consequences of consumption, and exploring their potential in therapeutic applications, Ipomoea plants remain a subject of fascination and scrutiny in the realm of phytopharmacology.

### 3.11. Nicotiana tabacum (Tobacco)

#### 3.11.1. Historical Background

*Nicotiana tabacum*, commonly known as tobacco, is a perennial herbaceous plant belonging to the Solanaceae family. It is an allotetraploid plant that evolved approximately 200,000 years ago through interspecific hybridization between the ancestors of *Nicotiana sylvestris* and *Nicotiana tomentosiformis* [235]. This plant species has been cultivated for centuries for recreational and narcotic purposes. The consumption trends of tobacco have evolved over time, with recreational use primarily through smoking, chewing, vaping, and narcotic use due to the presence of nicotine, which acts as a stimulant affecting the central nervous system [236].

#### 3.11.2. Drug Policy

The Tobacco Products Directive (TPD) was implemented by the European Union in 2014, and by 2021, all 27 Member States had adopted this law. Some regulations imposed on tobacco-related products include the requirement for e-cigarette leaflets with detailed information on usage, warnings, a maximum nicotine limit of 20 mg/mL, and child-resistant packaging. To date, following the implementation of this law, the number of neonatal and stillbirths caused by tobacco has dropped considerably by about 7%, demonstrating the importance of implementing such regulations worldwide [237].

#### 3.11.3. Mechanism of Action

The mechanism behind the narcotic and recreational effects of *Nicotiana tabacum* primarily lies in its chemical composition. Nicotine, an alkaloid predominantly found in tobacco leaves, is the primary compound responsible for the addictive properties of [238]. Additionally, other alkaloids present in tobacco, such as piperidine alkaloids, contribute to its physiological effects [239]. The presence of terpenes, heavy metals, and alkaloids in *Nicotiana tabacum* further adds to its chemical complexity and diverse effects on the human body [240].

Nicotine, the principal phytochemical in *Nicotiana tabacum*, is a well-known stimulant that affects various neurotransmitter systems in the brain, particularly the cholinergic system. It acts as an agonist at nicotinic acetylcholine receptors, leading to the release of neurotransmitters such as dopamine, norepinephrine, and serotonin. These neurotransmitters are associated with feelings of pleasure, alertness, and increased focus, contributing to the psychostimulant effects of nicotine [241]. Moreover, the pyrrolidine ring of nicotine plays a crucial role in its stimulant properties [242]. Nicotine, along with other alkaloids present in tobacco, influences the reward pathways in the brain, reinforcing the addictive nature of nicotine [243]. Additionally, the alkaloids contained in *Nicotiana tabacum* are known to have psychoactive effects that can enhance cognitive function, mood, and arousal, making tobacco a sought-after recreational plant [244].

#### 3.11.4. Pharmacological Properties and Health Implications

While *Nicotiana tabacum* has been widely consumed for recreational and narcotic purposes, it is crucial to acknowledge the adverse effects associated with its use. Tobacco consumption has been linked to various health issues, including but not limited to respiratory diseases, cardiovascular disorders, and an increased risk of cancer [245]. The addictive nature of nicotine also poses challenges for individuals trying to quit tobacco use, leading to long-term dependency and withdrawal symptoms upon cessation [244].

#### 3.11.5. Future Perspectives on Public Health

Looking toward the future, the implications of *Nicotiana tabacum* on public health remain a critical concern. With increasing awareness of the health risks associated with tobacco use, there is a growing need for comprehensive tobacco control measures to mitigate the negative impact on individuals and society as a whole. Public health campaigns, regulatory policies, and smoking cessation programs play a vital role in addressing the challenges posed by tobacco consumption [245].

#### 3.11.6. General Conclusion

Overall, *Nicotiana tabacum*, with its long historical significance and complex chemical composition, continues to be a plant of interest for both recreational and narcotic purposes. Understanding the mechanisms behind its effects, the chemical compounds responsible, the negative consequences of consumption, and the future implications for public health is essential in shaping policies and interventions to address the challenges associated with tobacco use. By synthesizing research findings and staying informed about the latest developments in the field, stakeholders can work toward promoting healthier lifestyles and reducing the burden of tobacco-related diseases on individuals and communities.

### 3.12. Mandragora officinarum (Mandrake)

#### 3.12.1. Historical Background

*Mandragora officinarum*, commonly known as Mandrake, is a perennial plant belonging to the Solanaceae family and is native to the eastern Mediterranean region. This plant has a rich historical significance, with mentions dating back to ancient times when it was used for various purposes, including medicinal and ritualistic practices. *Mandragora officinarum* contains potent active compounds such as scopolamine and atropine, which contribute to its narcotic and recreational effects [246]. The plant has been traditionally associated with mystical and magical properties, often linked to witchcraft and folklore due to its psychoactive properties [247].

Historically, the plant was used for its sedative and analgesic properties, often used in surgical procedures to relieve pain [248]. However, due to its potent effects and potential toxicity, the use of *Mandragora officinarum* for sedative and analgesic properties purposes has become less common in modern times. Nevertheless, there is still a niche interest in exploring the psychoactive potential of this plant, especially in alternative medicine practices [249].

#### 3.12.2. Mechanism of Action

In contemporary times, *Mandragora officinarum* continues to be of interest due to its potential for recreational and narcotic purposes. The plant has been studied for its chemical composition, with compounds like nolides being isolated, contributing to its pharmacological effects [250]. Additionally, alkaloids like atropine, scopolamine, and hyoscyamine have been identified in *Mandragora officinarum*, which are known for their psychoactive properties [251]. Alkaloids like atropine and scopolamine act on the central nervous system, producing sedative and hallucinogenic effects [251], while anolides have been associated with anti-inflammatory and neuroprotective properties, which may contribute to their recreational potential [250]. These compounds interact with various receptors in the body, leading to the psychoactive effects observed upon consumption of *Mandragora officinarum*.

#### 3.12.3. Pharmacological Properties and Health Implications

Despite its potential benefits, the consumption of *Mandragora officinarum* for recreational and narcotic purposes is not without risks. The plant contains potent compounds that can adversely affect human health, especially when consumed in high quantities or inappropriately. Studies have shown that prolonged use of *Mandragora officinarum* can lead to hepatotoxicity and other serious health complications. Additionally, the plant’s psychoactive properties can pose risks of addiction and overdose if not used cautiously [250].

#### 3.12.4. Future Perspectives on Public Health

Looking toward the future, the implications of *Mandragora officinarum* for public health remain a topic of concern. While the plant has a long history of traditional use, its recreational and narcotic potential raises questions about regulation and safety. Public health authorities need to be aware of the risks associated with the consumption of *Mandragora officinarum* and provide guidance on its responsible use. Further research is needed to understand better the pharmacological effects and toxicological profile of this plant to ensure the safety of individuals who may be inclined to use it for recreational purposes [251].

#### 3.12.5. General Conclusion

Overall, *Mandragora officinarum*, with its historical significance and potent chemical composition, presents a complex picture when it comes to its recreational and narcotic potential. While the plant has been traditionally revered for its mystical properties, its modern-day consumption trends raise important safety and public health considerations. Understanding the mechanisms through which *Mandragora officinarum* exerts its effects, the chemical compounds responsible for its psychoactive properties, the associated adverse effects of consumption, and the future implications for public health are crucial in navigating the use of this plant in recreational and narcotic contexts. Vigilance, regulation, and further research are essential to ensure that the recreational use of *Mandragora officinarum* is approached with caution and awareness of its potential risks.

Table 1 outlines the most commonly used plants for recreational and narcotic purposes, along with the associated phytocompounds. The table includes the plant’s scientific name, common name, plant family, used parts, and the specific phytocompounds responsible for the effects. This information provides a comprehensive overview of the botanical sources and chemical constituents utilized for recreational and narcotic purposes, aiding in understanding the pharmacological basis of these plants’ effects.

## 4. Popularity, Availability, Effects, and Risks of Medication and Substance Abuse

### 4.1. Popularity and Availability of Medication and Substance Abuse

The analysis of consumption patterns, coupled with the legislative developments surrounding psychoactive substances, provides valuable insights into the global prevalence and dissemination of these substances. The ramifications of the legalization of both medicinal and recreational cannabis remain ambiguous within the current discourse. Nevertheless, legalization has engendered a surge in market activity characterized by a proliferation of retail outlets [252]. Notably, in Canada, the pricing dynamics of recreational cannabis have been scrutinized. Research indicates that the cost of a gram of marijuana ranges from USD 5 to USD 14, with pricing differentials attributed to product quality and geographical location [253]. The affordability of cannabis has facilitated access for individuals from socioeconomically disadvantaged backgrounds. Initially, price hikes were contemplated as a deterrent to consumption; however, this strategy was rescinded to mitigate the incentivization of illicit, lower-priced cannabis products [254]. The proliferation of retail establishments has inadvertently fostered a misconception that cannabis consumption carries minimal risks [255]. Countries such as Spain and the Netherlands have adopted distinctive approaches to accommodate and regulate cannabis consumption. Spain has instituted Cannabis Social Clubs, which are dedicated to the legal cultivation and communal use of marijuana. Similarly, Amsterdam has established coffee shops that serve as venues for the controlled consumption of cannabis [256,257].

Tobacco, a widely consumed legal substance, is ubiquitously available globally and retailed in local convenience stores, gas stations, and specialized tobacco outlets [9,258]. The accessibility of tobacco products in these retail establishments poses a significant challenge, as their commercial presence exerts a direct influence on the behavior of smokers. The unrestricted availability of tobacco products serves as a potent temptation for individuals who smoke, thereby impeding efforts toward smoking cessation. Moreover, marketing strategies employed by tobacco companies often target vulnerable populations, strategically siting cigarettes in easily accessible locations frequented by the target demographic [259,260]. In addition to physical stores, tobacco products are equally prevalent online, further extending their reach to consumers. The marketing tactics utilized in online retail spaces have also played a pivotal role in shaping consumer preferences by introducing a variety of flavors to attract and retain customers. This diversification of product offerings through flavor enhancement has been instrumental in expanding tobacco companies’ customer base, thereby perpetuating tobacco product consumption [261].

When examining the landscape of opioid availability, it becomes apparent that these substances originate from diverse channels. They may be acquired through legitimate means, such as a valid prescription from a healthcare professional or illicit avenues involving unauthorized procurement of pain-relieving medications [262]. The dispensation of opioid prescriptions is typically reserved for cases where deemed essential, with established protocols in place to govern their appropriate administration. Nevertheless, individuals belonging to high-risk cohorts may engage in deceptive practices to manipulate prescribers into authorizing opioid medications, resorting to subterfuge if initial attempts prove unsuccessful, often resorting to illicit means of acquisition [263,264]. The dissemination of opioids in over-the-counter (OTC) formulations is notably observed in instances involving codeine and dextromethorphan [265]. Notably, products containing codeine have garnered significant popularity in various nations, including Italy, Poland, the United Kingdom, Spain, Germany, and the United States [266]. The literature documents instances of substance abuse and addiction stemming from the misuse of OTC medications, underscoring the potential risks associated with their unregulated availability [267]. Despite the widespread accessibility of these substances, certain countries have taken regulatory measures to curtail the sale of OTC products containing codeine, exemplified by Australia and France, in a bid to mitigate associated hazards [268].

Classified as a controlled substance, cocaine exhibits limited availability within the realm of illicit commerce. Noteworthy source nations implicated in the trade of this substance include Colombia, Mexico, and Afghanistan [269,270]. The production of narcotics transpires within these source countries, subsequently necessitating their transportation to intermediary transit nations. The market value of cocaine is intrinsically linked to its accessibility. Source countries, characterized by extensive manufacturing operations, typically offer this substance at a comparatively lower cost. Conversely, the price per kilogram escalates significantly, potentially up to a hundredfold, upon reaching the importing nations [270].

Understanding the multifaceted dimensions of substance availability is paramount in formulating effective interventions and policy changes to combat substance abuse. By delving into the various facets of substance availability, policymakers and stakeholders can gain a comprehensive insight into the intricate dynamics that underlie substance abuse trends. This nuanced understanding serves as a foundational basis for the strategic recalibration of existing laws and regulations, with the ultimate goal of curbing the prevalence of substance abuse within society. In the realm of substance abuse prevention and control, a thorough comprehension of the availability of different substances is indispensable. This comprehension extends beyond mere recognition of the physical presence of substances within communities to encompass a holistic grasp of the socioeconomic, cultural, and environmental factors that contribute to their accessibility. By dissecting the intricate web of factors that influence substance availability, policymakers can tailor interventions that address the root causes of substance abuse, thereby fostering more sustainable and impactful outcomes in the realm of public health and safety.

### 4.2. Effects and Risks of Medication and Substance Abuse

Substances of abuse can alter the consciousness of individuals who consume them. The psychoactive properties of these substances can elicit a state of euphoria [271]. For instance, cocaine exerts stimulant effects on the central nervous system, leading to heightened energy levels and enhanced cognitive focus [272]. Similarly, tobacco is recognized for its ability to induce a transient surge in energy [271]. In the case of cannabis, its consumption can impact motor coordination and cognitive judgment. Common experiences associated with marijuana use include feelings of anxiety, paranoia, and increased appetite [272,273].

Respiratory depression represents a critical adverse effect associated with opioid misuse, culminating in apnea that may ultimately lead to cardiac arrest. The onset of symptoms varies, typically manifesting within minutes following opioid administration, contingent upon the route of delivery. Concurrent administration of cocaine with opioids has been identified as a factor exacerbating the likelihood of overdose [274]. Hyperalgesia emerges as an unfavorable response to opioid therapy, predisposing individuals to addiction. This phenomenon may be misconstrued by both healthcare providers and patients as disease progression, prompting an escalation in dosage [68].

The cardiac implications of cocaine use can manifest acutely following ingestion or may become apparent following prolonged exposure. Acute cardiovascular manifestations of cocaine consumption include elevated blood pressure, increased heart rate, and, in severe instances, coronary artery spasms that can precipitate myocardial infarction. Chronic cocaine use is linked to a spectrum of cardiac complications, impacting various aspects of cardiac function, with non-ischemic cardiomyopathy emerging as a notable consequence. Research has established a correlation between cocaine abuse and conditions such as myocarditis, left ventricular hypertrophy, and heart failure [275,276]. The onset of the desired effects sought by users typically occurs within 5 min of administration, with peak effects typically observed around 30 min post-administration [272]. The morbidity and mortality rates among individuals who smoke are primarily attributed to cardiovascular diseases and cancer. Smoking-induced oxidative damage is identified as the primary etiological factor contributing to the development of various types of cancer. While lung cancer stands out as the most prevalent consequence, smoking significantly influences the incidence of cancer affecting the upper and lower gastrointestinal tract as well as the urogenital system [271,273].

The occurrence of sudden death among individuals using cannabis is likely associated with cardiovascular events [274]. Due to the legal restrictions on cannabis in certain regions, the body of research in this area remains somewhat inconclusive, although certain effects of cannabis use have been identified. Reports indicate that cannabis consumption can lead to impaired verbal and working memory, as well as potential alterations in brain function; however, the variability of these findings is influenced by factors such as the frequency of exposure and the concurrent use of substances like tobacco or cannabidiol [277]. Furthermore, the respiratory issues observed in cannabis users are often attributed to the combined use of tobacco and cannabis [278]. The effects of cannabis consumption typically manifest within approximately 10 min. Research has shown that detectable levels of tetrahydrocannabinol (THC) can persist in the bloodstream for up to 1–2 days in occasional users and up to 7 days in chronic users [276]. Following the cessation of cannabis use, individuals may experience a complex array of withdrawal symptoms encompassing both psychological and physical manifestations. Psychological symptoms may include insomnia, anxiety, depression, irritability, weight loss, aggression, and anger, while physical symptoms may manifest as fever, tremors, headaches, and sweating. These withdrawal symptoms typically emerge throughout one to two weeks and can onset as early as the first day of abstinence [277].

Table 2 presents the adverse reaction profile of the most frequently used substances of abuse. This table includes data on the various adverse effects associated with commonly abused substances such as opioids, cocaine, tobacco, and cannabis. The information provided in Table 2 points out the potential risks and harms associated with substance abuse. This information is valuable for developing interventions, treatment strategies, and harm reduction approaches to address substance abuse and its consequences.

## 5. Novel Insights on Substance Abuse Addiction

Substance abuse addiction is a significant global health issue, with a substantial burden on individuals and societies worldwide. According to the Global Burden of Disease 2010 Project (GBD), mental and substance use disorders contribute significantly to the burden of disease, with substance dependence being a complex disorder influenced by genetic and environmental factors [278,282]. The transition to parenthood has been identified as a novel mechanism of addiction vulnerability, highlighting the importance of understanding the neurobiology of addictive processes from a parenting-specific perspective [283]. Moreover, the modulation of the endocannabinoid system has been recognized as a vulnerability factor and a potential treatment target for stimulant addiction, emphasizing the role of neurobiological substrates in addiction [284].

Addiction is a multifaceted process characterized by neurobiological deficits that lead to a compulsive search for and use of addictive substances despite adverse consequences [285]. This compulsive behavior is underpinned by the reward-reinforcement circuit, which is implicated not only in addictive disorders but also in other psychiatric syndromes, highlighting the complex interplay of neurobiology in addiction and related behaviors [286]. The component model of addiction treatment targets enduring psychological, cognitive, and neurobiological vulnerabilities common to all addictive disorders, emphasizing the modifiability of these characteristics [279]. Substance abuse, including addiction, is a significant contributor to the global burden of disease, with substantial economic costs that are expected to increase significantly in the coming years [287].

Behavioral addictions, with their genetic and neurobiological aspects, are also crucial to recognize and understand to develop effective prevention and treatment strategies [288]. Epidemiological studies have shed light on the prevalence and patterns of substance abuse in different populations, such as college students, medical interns, and professional college students, highlighting the widespread nature of substance abuse across various demographics [280,289,290]. Socioeconomic disparities have been identified as influencing the uptake of substances of abuse, emphasizing the role of social factors in substance abuse behaviors [281].

The challenges faced by parents of youth abusing substances, such as non-adherence to medical instructions and violent behavior, underscore the complex social dynamics that contribute to substance abuse within families and communities [291]. Substance abuse not only affects individuals but also has broader implications for society, including increased levels of crime and misconduct associated with reduced self-control and increased impulsivity [292]. The prevalence of comorbidities associated with substance abuse, particularly opioid abuse, highlights the complex interplay between substance abuse and other health conditions, further emphasizing the multifaceted nature of addiction [293].

Neurobiological research has shown that substance abuse, particularly methamphetamine dependence, can lead to abnormalities in pituitary hormonal regulation, indicating the profound impact of substance abuse on physiological processes [294]. Additionally, the treatment of methamphetamine withdrawal with medications like methylphenidate and modafinil underscores the challenges associated with managing substance abuse and the need for effective pharmacological interventions to address withdrawal symptoms and cravings [295]. The elevated plasma prolactin levels observed in abstinent methamphetamine-dependent individuals further highlight the physiological changes that occur as a result of substance abuse [294]

Overall, substance abuse addiction is a complex and multifaceted issue with significant global implications. Understanding the neurobiological mechanisms underlying addiction is crucial for developing effective prevention and treatment strategies. By addressing the genetic, environmental, and social factors that contribute to substance abuse, it is possible to mitigate the burden of addiction on individuals and societies. Further research into the neurobiology of addiction and the development of targeted interventions are essential for combating the pervasive impact of substance abuse on public health.

### 5.1. Neurobiological Mechanisms of Substance Abuse Addiction

The brain’s reward system is integral to substance abuse addiction, involving a complex interplay of neurobiological processes that drive addictive behaviors. Addiction is characterized by dysregulation in motivational circuits, encompassing heightened incentive salience, habit formation, reward deficits, stress dysregulation, and compromised executive function. This dysregulation leads to significant neurobiological changes, including alterations in the reward system, overactivation of brain stress systems, and impaired prefrontal cortex function [295]. The addictive process disrupts stress and rewards neural circuits, shedding light on how substance use can impact caregiving processes [296].

The neurobiological foundation of addiction centers around the mesocorticolimbic dopamine system, where addictive substances elevate dopamine neurotransmission, attributing incentive salience to drug-related cues and contexts. Prolonged exposure to these substances induces enduring adaptations in the brain’s reward pathways, heightening sensitivity to drugs and associated cues [297]. Moreover, irregularities in dopamine regulation in the prefrontal cortex contribute to addictive behaviors, enhancing motivational value and diminishing control over substance use [298].

Understanding drug craving is pivotal in comprehending addiction, with clinical neurobiological research elucidating the neurobiological basis of craving and its role in addiction’s pathophysiology. Drug craving is linked to alterations in dopamine signaling and compulsive drug-seeking behavior, emphasizing the intricate connection between neurobiology and addictive behaviors [299]. Furthermore, addiction-induced dysregulation of stress and reward neural circuits can impact caregiving processes [300].

Genetic factors also significantly influence addiction, with specific genes impacting various aspects of addiction neurobiology, such as anxiety, impulsivity, and reward processing [301]. Common neurobiological vulnerabilities between addiction and other psychiatric disorders, like ADHD, highlight the shared neurobiology of these conditions, particularly in terms of impulsivity, reward sensitivity, and control deficits [300]. Pharmacotherapy targeting neurobiological alterations associated with addiction, including withdrawal symptoms, craving, and aberrant reward processing, offers a promising avenue for treating substance use disorders [302].

Substance dependence also has an underlying genetic influence, demonstrated mainly through twin and adoption studies. For example, suppose one sibling is addicted to alcohol, cannabis, cocaine, or other drugs. In that case, the risk of other siblings developing the same addictions is increased, with a prevalence rate of up to 80% [303].

The inheritance of addiction is determined by the interaction of several genes, known as polymorphisms, which can influence the organism’s normal functioning through changes in the DNA sequence. The most common and well-known polymorphisms are single-nucleotide variations (SNPs), which occur when one base is replaced by another, contributing to an increased risk of developing certain conditions, including addiction [304]. Genome-wide association studies (GWAS) are performed to explore genetic variants (SNPs) and to identify links between these variants and specific traits or diseases [305].

To explore the connection between nicotine addiction and genetic influence, a genome-wide association study (GWAS) focused on nicotinic acetylcholine receptor genes was conducted. The results indicated that the regions involved were chromosome 15 with the CHRNA5-CHRNA3-CHRNB4 genes and chromosome 8 with the CHRNA6-CHRNB3 genes. These regions were further analyzed by correlating single-nucleotide polymorphisms (SNPs) with the average daily number of cigarettes, thus demonstrating excessive smoking [303].

For other substances of abuse, such as cocaine, the genes involved in addiction are located on chromosomes 9 and 12. For opioids, dopaminergic genes and chromosome 14q or 17 are associated with excessive use. For cannabis, withdrawal syndrome is associated with chromosomes 1, 3, 6, 7, 9, 16, and 19 in the context of addiction [303,306].

Another important factor in the development of addiction is heritable changes in genomic phenotypes, which DNA does not directly determine. These changes, such as acetylation, phosphorylation, and methylation at the chromatin level, are known as epigenetics and are triggered by drug use [307,308].

DNA methylation plays a significant role in genetic imprinting. It involves adding a methyl group on cytosine (5-mC), inhibiting gene transcription. DNA methylation is carried out by DNA methyltransferases (DNMTs), and DNMT3a plays a crucial role in the postnatal brain. In former heroin users, excessive methylation of the OPRM1 promoter varies between ethnic groups, reducing binding to transcription factors such as Sp1. Maternal exposure to opioids may influence offspring behavior and morphine sensitivity, suggesting a possible epigenetic transmission [308].

Acetylation of histones reduces electrostatic interactions with DNA, allowing chromatin to relax and making DNA more easily accessible. Acetylation of histones H3 and H4, regulated by histone acetyltransferases (HATs) and CREB-binding proteins (CBPs), plays a role in the cocaine response. These histone changes influence the transcriptional activity of genes involved in drug-induced behaviors, including in brain areas such as the nucleus accumbens (NAc) and prefrontal cortex (PFC) [307].

The brain’s reward system intricately links to substance abuse addiction, with dysregulation in motivational circuits, dopamine signaling, and stress pathways contributing to the onset and perpetuation of addictive behaviors. Understanding the neurobiological foundations of addiction, encompassing dopamine, stress systems, and genetic influences, is crucial for developing effective interventions and treatments for individuals grappling with substance abuse disorders [309].

The neurobiological basis of substance abuse addiction involves intricate mechanisms that encompass sensitization, tolerance, and neuroplasticity. Sensitization and tolerance are adaptive responses of the brain to repeated exposure to drugs of abuse, leading to changes in the brain’s reward circuitry. Tolerance often develops to the pleasurable effects of substances like cocaine and heroin, prompting individuals to increase their dosage and exacerbating the molecular changes that underlie addiction [310]. On the other hand, neuroplasticity refers to the brain’s ability to reorganize itself by forming new neural connections. It plays a crucial role in addiction by mediating the highly efficient and stable memory abnormalities associated with substance abuse [311].

The neural rejuvenation hypothesis of cocaine addiction posits that repeated exposure to drugs induces plasticity mechanisms within the brain’s reward circuitry, leading to stable memory abnormalities characteristic of addiction [312]. This hypothesis underscores the role of neuroplasticity in shaping the addictive processes. It highlights how drug-induced changes in the brain’s structure and function contribute to the persistence of addictive behaviors. Understanding the molecular mechanisms underlying substance abuse is essential for developing effective therapeutic strategies to combat addiction [313].

Studies have shown that addiction involves a series of neuroplastic changes in the mesolimbic reward pathway, emphasizing the role of neuroplasticity in the development and maintenance of addictive behaviors [314]. Neuroplasticity in cholinergic neurons of the laterodorsal tegmental nucleus has been implicated in the development of cocaine addiction, underscoring the intricate interplay between different brain regions involved in reward processing and reinforcement learning. These findings highlight the complex neural adaptations that occur in response to drug exposure and how they contribute to the addictive process [315].

Drug-induced neuroplasticity, characterized by changes in neurotransmitter systems such as serotonin, glutamic acid, dopamine, and gamma-aminobutyric acid, plays a pivotal role in the pathophysiology of addiction [316]. The dysregulation of these neurotransmitter systems due to repeated substance abuse leads to maladaptive changes in the brain’s reward circuitry, contributing to the compulsive drug-seeking behaviors observed in addiction. Addiction is increasingly recognized as a neurobiological disorder that disrupts normal brain circuitry, leading to drug-induced neuroplastic changes that perpetuate addictive behaviors [317].

The role of hippocampal neurogenesis in addiction highlights the impact of addictive drugs on the brain’s ability to generate new neurons, with addictive substances suppressing neurogenic proliferation in the hippocampus. These neuroadaptive processes within motivation circuits regulated by the hippocampus contribute to the development of addictive behaviors and underscore the importance of understanding the neurobiological basis of addiction [318]. Cell adhesion molecule genes, particularly CDH13, have been implicated in regulating neuroplasticity in addiction, emphasizing the genetic and molecular underpinnings of addictive behaviors [319].

MeCP2, a protein involved in gene regulation, has been implicated in controlling brain-derived neurotrophic factor expression and cocaine intake, suggesting a role in the neurobiological mechanisms of addiction [320]. Distress tolerance has been identified as a predictor of early treatment dropout in substance abuse facilities, highlighting the psychological factors that influence treatment outcomes in addiction [321]. Furthermore, brain anatomy alterations associated with social networking site addiction underscore the impact of excessive behaviors on brain morphology and neuroplasticity [322].

Addiction neuroscience aims to uncover the neural basis of addiction by mapping changes in the brains of individuals with substance use disorders, shedding light on the neurobiological underpinnings of addictive behaviors [323]. Psychedelic-induced neuroplasticity has also been studied in the context of addiction, emphasizing the role of neuroplastic changes in dopaminergic neurons in driving addictive phenomena [324]. Neuromodulation therapies targeting specific brain regions have shown promise in reducing relapse in alcohol addiction, highlighting the potential of neuroscientific interventions in addiction treatment [325].

The dysregulation of prefrontal cortical control over the nucleus accumbens core due to high-fat diets has been implicated in triggering drug-seeking behaviors and relapse, underscoring the role of neuroplastic changes in addiction [326]. GABA receptor modulators have been shown to affect neuroadaptation to alcohol and cocaine, suggesting a link between GABAergic neurotransmission and addictive behaviors [327]. Addiction is increasingly viewed as a disorder of experience-dependent neuroplasticity, emphasizing the role of synaptic remodeling in reward and motivation circuits in driving addictive behaviors [328].

Overall, the neurobiological basis of substance abuse addiction involves a complex interplay of sensitization, tolerance, and neuroplasticity mechanisms that underlie the development and persistence of addictive behaviors. Understanding how drugs of abuse affect the brain’s reward circuitry, neurotransmitter systems, and gene regulation is crucial for developing effective interventions to combat addiction. By unraveling the molecular and neural processes involved in addiction, researchers can pave the way for innovative treatment strategies that target the root causes of substance abuse disorders.

Substance abuse has a significant impact on brain function and behavior, leading to cognitive impairment, emotional dysregulation, behavioral changes, and the progression from substance use to addiction. Chronic alcoholism, for example, is linked to cognitive deficits affecting memory, attention, and decision-making [329]. Individuals with substance use disorders commonly experience emotional dysregulation, resulting in mood swings, anxiety, and depression [330]. This emotional dysregulation can contribute to risky behaviors such as compulsive drug seeking and relapse [331]. The transition from substance use to addiction involves intricate neurobiological processes, including the development of craving and dependence [332].

Research has extensively documented the impact of substance abuse on cognitive function, emphasizing the cognitive deficits seen in individuals with alcohol use disorders [329]. These deficits can affect various cognitive domains, including memory, attention, and executive functions, ultimately influencing decision-making abilities. Substance abuse can lead to structural changes in the brain that worsen cognitive impairments, particularly in areas related to impulse control. Chronic substance abuse not only acutely affects cognitive function but also contributes to long-term brain dysfunction, perpetuating impulsive behaviors and cognitive deficits [333].

Emotional dysregulation is a common consequence of substance abuse, with individuals often experiencing mood swings, anxiety, and depression [331]. This emotional dysregulation can be a maladaptive coping mechanism, leading to impulsive behaviors and risky decision-making. Studies have highlighted the significant role of emotional dysregulation in the development of various psychiatric disorders, including substance use disorders [331]. Additionally, childhood abuse has been associated with emotion dysregulation in adulthood, heightening the risk of substance abuse and related behavioral issues [330].

Behavioral changes linked to substance abuse encompass compulsive drug seeking and relapse, driven by alterations in brain function and reward pathways. Individuals with substance use disorders frequently display compulsive behaviors aimed at acquiring and using drugs despite negative consequences. These behavioral changes are closely tied to the neurobiological basis of addiction, involving modifications in the brain’s reward circuitry and the development of tolerance [332]. Furthermore, substance abuse can lead to changes in brain regions responsible for impulse control, further fueling compulsive drug-seeking behaviors [333].

The transition from substance use to addiction involves a complex interplay of neurobiological factors, including craving and dependence. Craving, characterized by an intense desire for a substance, is a key feature of addiction and is associated with changes in brain regions involved in reward processing. Dependence reflects the body’s adaptation to the substance, resulting in withdrawal symptoms upon cessation of use. The neurobiological underpinnings of craving and dependence underscore the chronic nature of addiction and the difficulties in achieving and maintaining sobriety [332].

In summary, substance abuse has a multifaceted impact on brain function and behavior, encompassing cognitive impairment, emotional dysregulation, behavioral changes, and the progression from substance use to addiction. Chronic substance use disorders are associated with cognitive deficits, emotional dysregulation, and compulsive behaviors driven by alterations in brain function and reward pathways. Understanding the neurobiological basis of substance abuse is crucial for developing effective interventions targeting cognitive, emotional, and behavioral aspects of addiction.

### 5.2. Treatment Approaches and Interventions Regarding Substance Abuse Addiction

Substance abuse addiction is a multifaceted issue that necessitates a comprehensive approach for effective treatment. Various interventions, such as pharmacological, behavioral, psychotherapeutic, and emerging therapies, are crucial in addressing substance abuse addiction. Pharmacological interventions involve the use of medications to manage addiction and withdrawal symptoms [334]. These medications target different aspects of addiction, such as reducing cravings or blocking the effects of drugs, to support individuals in their recovery process. Behavioral therapies, including cognitive-behavioral therapy (CBT) and Motivational Interviewing, are widely recognized as practical approaches to treating substance abuse addiction. CBT helps individuals identify and change negative thought patterns and behaviors associated with substance abuse. At the same time, Motivational Interviewing aims to enhance motivation and commitment to change addictive behaviors [335,336].

Psychotherapeutic approaches are essential in addressing underlying mental health issues that may contribute to substance abuse addiction [337]. Individuals with co-occurring disorders, such as substance use disorder and posttraumatic stress disorder, benefit from integrated treatment that addresses both conditions simultaneously. Emerging therapies, including neurostimulation and gene therapy, offer innovative ways to target the neurological mechanisms involved in addiction. These therapies hold promise in advancing treatment strategies for addictions and dual-diagnosis disorders by enhancing adaptive regulation of brain functions [317].

Research has shown that cognitive bias modification and cognitive control training are valuable tools in addressing addiction and related psychopathology [338]. These interventions target cognitive processes contributing to addictive behaviors, helping individuals develop healthier thinking patterns and decision-making skills. Additionally, cognitive-behavioral therapy has been identified as a leading behavioral approach for treating alcohol and other drug use disorders. By addressing maladaptive thoughts and behaviors, CBT equips individuals with coping strategies to manage triggers and prevent relapse [335].

Furthermore, motivational variables and interventions play a significant role in treatment outcomes for addictive behaviors [339]. Motivation for treatment is a crucial factor in determining engagement, compliance, and success in overcoming addiction. Patient-centered approaches, such as those based on motivational interviewing, emphasize the importance of aligning treatment goals with the individual’s preferences and values. By involving patients in decision-making and goal-setting, treatment interventions can be more tailored and effective in supporting long-term recovery [340].

In the realm of psychotherapy, interventions like cognitive-behavioral therapy have demonstrated efficacy in treating substance use disorders, particularly when combined with motivational strategies [341]. These evidence-based interventions provide individuals with the skills and support needed to address addictive behaviors and maintain sobriety. Additionally, coping strategy training, as part of cognitive-behavioral therapy, helps individuals develop effective ways to manage stressors and cravings that may lead to relapse [342].

In addition to cognitive-behavioral therapy (CBT), systemic therapy is another effective treatment for substance dependence. The systemic treatments addressed for substance abuse date back as far as the 1986s and are currently making a significant impact in addiction treatment. Systemic addiction treatment is structured in five levels. At level 1, the family is not involved, and therapy is isolated. At level 2, the counselor educates and advises the family, often recommending family therapy. At level 3, the counselor works more directly with the family, helping each member cope. Level 4 involves analyzing and intervening in dysfunctional family patterns. Family therapy for systemic addiction treatment is only implemented at level 5, and from this point on, interventions become more complex. Over the years, the best results in reducing substance use have been achieved in treatment settings in which family and life partners have been actively involved [343].

The life partners of those who are substance abusers are most strongly affected by addictive behavior. Despite their best efforts to help them overcome this behavior, the people close to them often refuse to offer help because of helplessness and disobedience. In these situations, psychologists recommend that the couple participate in motivational interviewing (MI), helping them analyze their behavior and find and introduce the motivation to change, thus overcoming difficult moments together [344].

If the addicted person is in a stable relationship in which neither partner has a co-existing psychiatric condition, behavioral couple therapy (BCT) is recommended for about 20 weeks. During this time, the partner supports the partner in trying to help stop substance use and strengthen the relationship. In solution-focused therapy (SFT), patients are encouraged to discuss their problems or concerns openly. With the therapist’s help, they identify solutions and set goals to improve their hope for change. Research shows that 36% of patients who attend at least two SFT sessions can improve their behavior [344].

Another type of systemic therapy is multisystemic family therapy (MFT). Family members play a crucial role in the patient’s recovery process, offering help by providing an environment that keeps them away from the negative influences of substances. The family will be assigned specific tasks to address the current problems so they can work together daily and weekly to accomplish them, one step closer to solving the central issue. Galanter and colleagues evaluated the effectiveness of network therapy compared with individual counseling; both included in a buprenorphine maintenance protocol. The results showed a significantly higher rate of opioid abstinence in the group receiving network therapy (about 65%) compared with those receiving individual therapy (about 45%) [3,4]. Because the family has a significant role during this time in treatment, the Center for Substance Abuse Treatment classifies this approach as level 3. Level 2 is the Johnson-style intervention, in which for 4 weeks, family and friends of the addicted person are taught how to encourage him/her in the treatment process. During the meeting, the patient explained in a calm, caring, and supportive tone how his relationships with everyone close to him had changed negatively [345,346].

Nowadays, the number of teenage substance users is increasing, so addiction treatment is also very vital. One of the approaches is SOFT therapy or strengths-oriented family therapy, in which, for 2 h, adolescents participate in family groups, where each member’s strengths are assessed and used to solve problems. In the case of runaway adolescents, Ecological Family Therapy (EBFT), where each family member meets with the adolescent in separate sessions to discuss the factors that led to the runaway, after which the therapist helps the family to keep the adolescent at home [347]. Another treatment approach is multisystemic therapy (MST), which involves the family providing support at school and in circles of friends and community. Initially, with the help of the parents, the therapist will gather information on how the adolescent integrates substances with daily activities, thus determining how to approach the problem. The progress of the therapy will be evaluated over a maximum period of 6 months, with the help of unique forms that mention all the behavioral and communication changes that the parents should gradually introduce in the relationship with the adolescent [347,348].

Occupational therapy services have also been recommended for individuals with substance abuse disorders, highlighting the importance of a holistic and interdisciplinary approach to treatment [349]. By addressing the functional aspects of daily living and promoting meaningful activities, occupational therapists can support individuals in their recovery journey. Moreover, interventions tailored to specific stages of change and coping skills acquisition are essential for individualized treatment approaches in addiction therapy. Understanding where individuals are in their readiness to change and equipping them with coping mechanisms can enhance treatment outcomes and reduce the risk of relapse [350].

Substance abuse addiction is a complex issue affecting individuals globally. The misuse of substances like alcohol, opioids, and tobacco poses significant health risks and societal burdens. Addressing substance abuse addiction requires a comprehensive approach that includes pharmacological interventions aimed at mitigating the negative effects of these substances and supporting individuals in their recovery journey [351].

Alcohol is a commonly abused substance, with medications like acamprosate, naltrexone, and disulfiram used in the treatment of alcohol use disorder. Acamprosate reduces alcohol cravings by acting on glutamate receptors, while naltrexone blocks opioid receptors, diminishing the rewarding effects of alcohol consumption. Disulfiram induces an unpleasant reaction when alcohol is consumed, acting as a deterrent [352].

Opioids are another class of substances commonly associated with addiction. Medications such as methadone and buprenorphine are utilized in opioid addiction treatment. Methadone reduces withdrawal symptoms and cravings by acting on opioid receptors, while buprenorphine, a partial opioid agonist, helps manage cravings without producing the same high as full opioids [352].

Tobacco use disorder is prevalent among individuals with substance abuse issues. Smoking cessation interventions, including counseling and pharmacotherapy, are crucial in supporting individuals undergoing substance abuse treatment. Providing quit-smoking medications as part of substance abuse treatment programs can significantly enhance outcomes and reduce the overall burden of tobacco-related health issues [353].

Integrating primary medical care with addiction treatment has been shown to be beneficial for individuals with substance abuse and medical comorbidities. This integrated approach not only improves health outcomes but also proves to be cost-effective in the long run. By addressing both addiction and underlying medical conditions simultaneously, individuals receive comprehensive care that addresses their holistic needs [354].

Psychotropic drugs targeting various neurotransmitter systems in the brain, such as GABA, glutamate, dopamine, and opioids, play a crucial role in the pharmacological treatment of substance abuse disorders. These medications act on specific receptors to modulate the effects of addictive substances, helping individuals manage cravings, withdrawal symptoms, and the reinforcing effects of drugs [355].

Faith-based interventions have also been explored in the treatment of substance addiction, emphasizing a holistic approach that includes biological, psychological, environmental, and spiritual aspects of recovery. Such interventions often incorporate detoxification strategies, counseling, skill training, and social support alongside religious and spiritual growth to aid in the recovery process [356].

In the realm of addiction treatment, primary care physicians play a pivotal role in recognizing addiction, providing interventions for intoxication and withdrawal, and offering long-term care for individuals with substance use disorders [357]. Brief interventions by primary care providers can have a significant impact on early intervention and treatment initiation for substance abuse, leading to improved outcomes and reduced societal costs associated with addiction [358].

The pharmacological treatment approaches for substance abuse addiction encompass a diverse range of medications targeting different substances and neurotransmitter systems in the brain. From alcohol and opioids to tobacco and other illicit substances, medications play a crucial role in managing cravings, withdrawal symptoms, and the reinforcing effects of drugs. Integrating primary medical care and the expertise of primary care physicians further enhances the effectiveness of addiction treatment programs, offering individuals comprehensive support on their path to recovery [352,354].

Overall, the treatment of substance abuse addiction requires a comprehensive and integrated approach that combines pharmacological, behavioral, psychotherapeutic, and emerging therapies. By addressing the biological, psychological, and social aspects of addiction, individuals can receive tailored interventions that support their recovery and long-term well-being. Through evidence-based practices, patient-centered care, and interdisciplinary collaboration, the field of addiction treatment continues to evolve to meet the diverse needs of individuals struggling with substance abuse.

## 6. Advancements in Technology for Detection of Substance Abuse

Substance abuse detection is a crucial aspect of addressing the challenges posed by drug misuse and addiction. Various methodologies and devices have been developed to identify the presence of substances in individuals. One common approach involves using immunologic techniques in analyzing bodily fluids like sweat and saliva. These techniques have been instrumental in detecting a wide range of substances, including illicit drugs, providing a non-invasive and efficient means of screening individuals for substance abuse. Additionally, advanced analytical methods such as thin-layer chromatography (TLC), high-performance liquid chromatography (HPLC), and capillary electrophoresis (CE) have been employed for substance abuse detection, offering high sensitivity and specificity in identifying different substances [359].

Substance abuse encompasses the misuse of various drugs, including marijuana, alcohol, cocaine, heroin, and cigarettes [360]. Detection methods tailored to these specific substances are crucial in effectively identifying and addressing substance abuse issues. For instance, in the context of sports, detecting the abuse of growth hormones presents unique challenges, but utilizing discriminant functions based on multiple markers has shown promise in enhancing sensitivity and specificity in detecting growth hormone abuse. This highlights the importance of employing specialized detection methodologies for different substances to ensure accurate and reliable results [361].

Moreover, the prevalence of substance abuse extends to different populations, including older adults, where brief interventions by primary care providers have been shown to significantly impact preventing medical morbidity and improving quality of life [362]. Tailoring detection methods to suit the needs of specific demographics, such as older individuals, is essential in addressing substance abuse effectively. Additionally, the use of biochip array technology has emerged as a highly sensitive and specific technique for detecting substances of abuse in hair samples, offering a valuable tool in substance abuse detection [363].

The incidence of substance abuse has exhibited a notable escalation in recent years, prompting a corresponding surge in efforts to detect these substances. Detection methodologies predominantly rely on metabolite analysis, a delicate process owing to the higher concentration of these metabolites in urine compared to blood. Conventional blood and urine screening methods have proven inadequate for precise determinations. They are susceptible to cross-reactivities [359,364].

One of the most effective methodologies for detecting substances such as nicotine and opioids is rooted in immunological principles, involving the development of antibodies and vaccines tailored to target these specific compounds [359]. These vaccines stimulate the production of high-quality antibodies that work to counteract the presence of the substances in the bloodstream. By utilizing this vaccine, the ability of the drug to traverse the blood–brain barrier is impeded, consequently mitigating the majority of the pharmacodynamic responses associated with these substances. While initial iterations of these vaccines, particularly those designed against nicotine, yielded unremarkable outcomes in clinical trials, subsequent generations have demonstrated significantly improved efficacy in preclinical models [359,365]. Furthermore, biochemical analysis can provide insights into nicotine usage. Carbon monoxide (CO) levels can serve as an indicator of recent tobacco smoke exposure from various sources such as pipes, cigarettes, hookahs, and cigars. The assessment of CO concentrations takes into account the typical environmental exposure of patients, with any elevation exceeding 1–2 parts per million (ppm) surpassing the endogenous production of CO within the human body [366].

Detection methods that exhibit high sensitivity toward minute sample volumes have gained significant attention, particularly within the realm of opioid detection. Among these methods, biosensors utilizing nanomaterials have emerged as a prominent approach. Nanobiosensors feature a nanostructure serving as a signal transducer, with a biological recognition molecule being incorporated onto the surface of this nanostructure. Notably, optical sensor systems utilizing nanomaterials, such as colorimetry and fluorescence-based methods, have been extensively explored for detection purposes. Gold and silver nanoparticles are commonly employed in these systems due to their capacity to induce Surface Plasmon Resonance [365,367].

The identification of illegal substances poses a significant challenge in various settings, including schools and institutions. One potential method for detection involves the analysis of sewage water to assess the prevalence of illicit drugs. A recent study conducted in Slovakia aimed to investigate the occurrence of drugs within school environments. The researchers employed solid-phase extraction combined with direct injection combined ultra-performance liquid chromatography-tandem mass spectrometry to analyze the samples. The findings from this study revealed the presence of several biomarkers, with notable detections, including those specific to nicotine, cannabis, morphine, and codeine [368].

In the context of cannabis detection, tetrahydrocannabinol (THC) serves as the primary psychoactive component of interest. Various biological samples such as urine, blood, and saliva are commonly utilized for THC analysis in laboratory settings. Noteworthy advancements in THC detection methods include the utilization of colorimetric electrochemical affinity biosensors, giant magnetoresistive biosensors, competitive volumetric bar-chart chip assays, dye-displacement assays employing aptamers, as well as colorimetric and fluorescence-based lateral flow assays [369,370].

In the context of roadside testing conducted on drivers for the identification of cannabis use, superior levels of specificity, sensitivity, and accuracy were observed in devices capable of detecting lower concentrations of THC, as opposed to those designed to identify higher levels of this compound [371].

The field of substance abuse detection continues to evolve with advancements in technology and methodologies. Various detection tools are available for identifying abused substances, from immunologic techniques and chromatography methods to biochip array technology. Tailoring detection methods to specific substances and populations, such as athletes, teenagers, older adults, and individuals with co-occurring disorders, is crucial in effectively addressing substance abuse. By integrating substance abuse detection into healthcare practices, counseling programs, and educational curricula, professionals can play a vital role in combating substance abuse and promoting overall well-being in individuals affected by drug misuse.

## 7. Conclusions

In the present article, a thorough investigation into the historical, societal, and scientific aspects of medication, substance abuse, and plant use for recreational and narcotic purposes was conducted. The review delved into the early utilization of plants for such purposes, the evolution of laws and regulations surrounding these substances, and the profound impact that medication and substance abuse have had on society. Furthermore, the article explored the most commonly used plants for recreational and narcotic purposes in contemporary times. The popularity, availability, effects, and risks associated with these substances were meticulously examined, shedding light on the complex interplay between human behavior, societal norms, and pharmacological effects.

The review article also delved into novel insights on substance abuse addiction, elucidating the neurobiological mechanisms that underlie addiction to substances. By exploring the intricate pathways in the brain that are involved in addiction, the article provided a deeper understanding of why individuals may become dependent on substances and the challenges they face in overcoming addiction. Additionally, the review discussed various treatment approaches and interventions for substance abuse addiction, highlighting the importance of a multidisciplinary approach that integrates pharmacological, psychological, and social interventions to address the complex nature of addiction. Moreover, advancements in technology for the detection of substance abuse were explored, showcasing how innovative tools and techniques are being developed to identify and monitor substance abuse more effectively.

Further research in this field is essential to deepen our understanding of the complexities of medications, substance abuse, and plant use for recreational and narcotic purposes. Studies should focus on elucidating the mechanisms underlying addiction, exploring novel treatment modalities, and developing targeted interventions to address substance abuse effectively. Additionally, education plays a crucial role in raising awareness about the risks associated with substance abuse and promoting responsible use of medications and plants for recreational purposes. By disseminating accurate information and fostering a culture of informed decision-making, education can empower individuals to make healthier choices and reduce the prevalence of substance abuse in society.

The implications of the findings presented in the current paper are far-reaching, with implications for public health, policy-making, and clinical practice. By recognizing the complex interplay between biological, psychological, and social factors in substance abuse, policymakers can develop more effective strategies to prevent and treat addiction. Healthcare providers can also benefit from a deeper understanding of the neurobiological mechanisms of addiction, enabling them to tailor treatment approaches to individual patients’ needs. Furthermore, the insights gleaned from advancements in technology for detecting substance abuse can inform the development of more sensitive and specific screening tools, enhancing early intervention and support for individuals struggling with addiction.

In light of the complexities surrounding medications, substance abuse, and plant use for recreational and narcotic purposes, several recommendations can be made to improve substance abuse management. Firstly, there is a need for enhanced collaboration between researchers, healthcare providers, policymakers, and community stakeholders to address substance abuse comprehensively. By fostering interdisciplinary partnerships, a more holistic approach to substance abuse prevention and treatment can be developed, integrating insights from various fields to create tailored solutions for individuals at risk of addiction.

Secondly, efforts should be made to destigmatize addiction and promote a compassionate and non-judgmental approach to individuals struggling with substance abuse. By reducing the stigma associated with addiction, individuals may be more inclined to seek help and engage in treatment, ultimately improving outcomes and reducing the burden of substance abuse on society. Education campaigns that raise awareness about the complexities of addiction and challenge misconceptions about substance abuse can play a pivotal role in shifting societal attitudes toward a more empathetic and supportive stance.

Thirdly, the development of evidence-based interventions that target the underlying mechanisms of addiction is crucial for improving substance abuse management. By investing in research that elucidates the neurobiological pathways involved in addiction, novel treatment modalities can be developed that address the root causes of substance abuse and promote long-term recovery. Additionally, the integration of technology-driven approaches for detecting substance abuse can enhance early intervention and monitoring, enabling healthcare providers to intervene proactively and support individuals in their recovery journey.

Overall, the article provides a comprehensive overview of the historical, societal, and scientific dimensions of substance use and abuse. By synthesizing a wealth of information on medications, substance abuse, and plant use for recreational and narcotic purposes, the article sheds light on the multifaceted nature of addiction and the challenges it poses to individuals and society at large. The key points highlighted in the article underscored the historical roots of substance use, the evolving legal landscape surrounding these substances, the societal implications of substance abuse, and the pharmacological effects of various plants used for recreational purposes. By examining the most commonly used plants for recreational and narcotic purposes, the review sheds light on the diverse array of substances that individuals may encounter in today’s society, each with its unique effects and risks. Further research, education, and collaboration are essential to address the complexities of substance abuse effectively and promote healthier outcomes for individuals struggling with addiction.

## Figures and Tables

**Table 1:** The most commonly used plants for recreational and narcotic purposes.

Latin Name	Common Name	Family	Used Part(s) of the Plant	Phytocompound(s)	References
*Cannabis sativa*	Cannabis	Cannabaceae	Flowering tops, leaves	Δ9-tetrahydrocannabinol (THC), cannabidiol (CBD)	[100]
*Erythroxylum coca*	Coca plant	Erythroxylaceae	Leaves	Cocaine	[137]
*Papaver somniferum*	Opium poppy	Papaveraceae	Dried latex from seed pods	Morphine, codeine, thebaine	[128]
*Mitragyna speciosa*	Kratom	Rubiaceae	Leaves	Mitragynine, 7-hydroxy mitragynine	[93]
*Catha edulis*	Khat	Celastraceae	Leaves stems	Cathinone, cathine	[150]
*Salvia divinorum*	Holy sage	Lamiaceae	Leaves	Salvinorin A	[167]
*Lophophora williamsii*	Peyote	Cactaceae	Dried top of the cactus	Mescaline	[179]
*Banisteriopsis caapi*	Soul vine	Malpighiaceae	Stems, bark, leaves	Harmine, harmaline	[192]
*Datura stramonium*	Devil’s trumpet	Solanaceae	Leaves, seeds	Scopolamine, atropine	[207]
*Ipomoea* sp.	Morning glory	Convolvulaceae	Seeds	Ergine (lysergic acid amide)	[229]
*Nicotiana tabacum*	Tobacco	Solanaceae	Leaves	Nicotine	[242]
*Mandragora officinarum*	Mandrake	Solanaceae	Roots	Atropine, scopolamine	[247]

**Table 2:** Adverse reactions profile and receptor targets of frequently abused substances.

Substance Abuse	Adverse Effects	Receptors	References
Opioids	Constipation		[69,275]
	Respiratory depression		[69,275]
	Sedation	μ, δ, κ	[69,275]
	Addiction		[69]
	Hyperalgesia		[69]
	Hallucinations		[69]
Cocaine	Mydriasis		[273]
	Heart diseases	D2, D3	[273,279]
	Tremors	α1-adrenergic	[273,279]
	Hostile conduct	β2-adrenergic	[273]
	Insomnia	5-HT	[273]
	Disorientation		[273]
Tobacco	Coronary artery disease		[257]
	Atrial fibrillation		[257]
	Hypertension		[257,270]
	Dyslipidemia	nACH	[270]
	Chronic obstructive pulmonary disease		[280]
	Cancer		[280]
	Infertility		[281]
Cannabis	Cardiovascular events		[263]
	Cough		[273]
	Wheezing breath	CB1, CB2	[259,273]
	Emphysema		[2]
	Infertility		[272]
	Psychoactive effects		[274]

## Data Availability

The original contributions presented in the study are included. Further inquiries can be directed to the corresponding author.
